# Elevated [CO_2_] negatively impacts C_4_ photosynthesis under heat and water stress without penalizing biomass

**DOI:** 10.1093/jxb/erad063

**Published:** 2023-02-17

**Authors:** Yazen Al-Salman, Oula Ghannoum, Francisco Javier Cano

**Affiliations:** ARC Centre of Excellence for Translational Photosynthesis, Hawkesbury Institute for the Environment, Western Sydney University, Richmond, NSW, Australia; ARC Centre of Excellence for Translational Photosynthesis, Hawkesbury Institute for the Environment, Western Sydney University, Richmond, NSW, Australia; ARC Centre of Excellence for Translational Photosynthesis, Hawkesbury Institute for the Environment, Western Sydney University, Richmond, NSW, Australia; Instituto de Ciencias Forestales (ICIFOR-INIA), CSIC, Carretera de la Coruña km 7.5, 28040, Madrid, Spain; University of Birmingham, UK

**Keywords:** CO_2_ response, C_4_ photosynthesis, drought response, heat tolerance, leaf temperature, stomatal kinetics, sorghum

## Abstract

Elevated [CO_2_] (eCO_2_) and water stress reduce leaf stomatal conductance (*g*_s_), which may affect leaf thermoregulation during heat waves (heat stress). Two sorghum lines, with different leaf width were grown in a glasshouse at a mean day temperature of 30 °C, under different [CO_2_] and watering levels, and subjected to heat stress (43 °C) for 6 d at the start of the reproductive stage. We measured leaf photosynthetic and stomatal responses to light transients before harvesting the plants. Photosynthesis at growth conditions (*A*_growth_) and biomass accumulation were enhanced by eCO_2_ under control conditions. Heat stress increased *g*_s_, especially in wider leaves, and reduced the time constant of stomatal opening (*k*_open_) at ambient [CO_2_] but not eCO_2_. However, heat stress reduced photosynthesis under water stress and eCO_2_ due to increased leaf temperature and reduced evaporative cooling. eCO_2_ prevented the reduction of biomass under both water and heat stress, possibly due to improved plant and soil water status as a result of reduced *g*_s_. Our results suggest that the response of the C_4_ crop sorghum to future climate conditions depends on the trade-off between low *g*_s_ needed for high water use efficiency and drought tolerance, and the high *g*_s_ needed for improved thermoregulation and heat tolerance under an eCO_2_ future.

## Teaser

Elevated [CO_2_] reduced thermoregulation of sorghum leaves due to lower stomatal conductance, and exacerbated photosynthetic depression under water and heat stress.

## Introduction

Rising atmospheric carbon dioxide concentration ([CO_2_]), due to anthropogenic activity, is a driver of climate warming and increased frequency and intensity of extreme heat and drought events (IPCC, 2021). C_4_ crops, such as sorghum, are known to thrive under warm temperatures and to tolerate droughts, but the limits of their tolerance are still unclear especially under combined future stresses (Lobell *et al.*, 2013; Leakey *et al.*, 2019). Sorghum is a key crop in the tropical and subtropical regions of the world, where the impact of climate change on agricultural systems is expected to be intense (Watson-Lazowski and Ghannoum, 2021). C_4_ crops are generally characterized by high productivity and water use efficiency (WUE; defined as carbon gain per water use) (Long, 1999). Nevertheless, increasing demand for food worldwide under changing climates requires the development of more tolerant varieties, and a greater understanding of how C_4_ crops respond to environmental stresses.

C_4_ photosynthesis is characterized by the operation of a CO_2_-concentrating mechanism (CCM), leading to the saturation of photosynthetic carbon assimilation rates (*A*_n_) at ambient CO_2_ (aCO_2_), and a weaker stimulation to elevated [CO_2_] (eCO_2_) than in C_3_ photosynthesis (Ghannoum *et al.*, 2000; Leakey, 2009). In contrast, eCO_2_ reduces stomatal conductance (*g*_s_), leading to improved intrinsic water use efficiency (iWUE, defined as *A*_n_/*g*_s_) in C_4_ plants (Conley *et al.*, 2001). Under well-watered (WW) conditions, eCO_2_ elicits little biomass stimulation in C_4_ plants (Ghannoum *et al.*, 1997, 2001; Ziska and Bunce, 1997; Wand *et al.*, 1999, 2001; Wall *et al.*, 2001), due to the minor photosynthetic stimulation (Kimball *et al.*, 2002; Leakey *et al.*, 2004; Long *et al.*, 2004).

Water stress (WS) reduces *g*_s_ and hence intercellular [CO_2_] (*C*_i_) of C_4_ leaves. At moderate WS, eCO_2_ increases *C*_i_ and maintains photosynthetic rates, which can drive growth (Conley *et al.*, 2001; Lecain *et al.*, 2003; Leakey *et al.*, 2004, 2006; Ghannoum, 2009). Additionally, eCO_2_ can enhance the biomass of C_4_ plants under WS due to reduced transpiration, which preserves soil water, allowing for continued plant growth (Samarakoon and Gifford, 1996; Seneweera *et al.*, 1998; Ghannoum *et al.*, 2000; Leakey *et al.*, 2004, 2006; Leakey, 2009; Kimball, 2016). Under severe WS, non-stomatal factors such as damage to the light-harvesting complex and reduced enzyme activity limit photosynthesis in a CO_2_-independent manner (Ghannoum *et al.*, 2003; Ghannoum, 2009; Tardieu *et al.*, 2018).

The third aspect of the future climate is the increase in extreme heat events (Alexander *et al.*, 2006). High air temperature (*T*_air_) increases the leaf-to-air vapour pressure deficit (VPD), which can promote stomatal closure to conserve water and lead to higher leaf temperature; *T*_leaf_ (Mott and Parkhurst, 1991; Shope *et al.*, 2008). Increased *T*_leaf_ below the thermal optimum (~35 °C for sorghum) can improve photosynthesis and leaf expansion rates in C_4_ crops (Ben-Haj-Salah and Tardieu, 1995; Long, 1999; Tardieu *et al.*, 1999; Massad *et al.*, 2007; Sonawane *et al.*, 2017). Increased *T*_leaf_ beyond optimum temperatures (+40 °C) damages the photosynthetic machinery and disrupts membrane stability (Siebke *et al.*, 2003; Sage and Kubien, 2007). Moreover, higher *T*_leaf_ promotes evaporation from the mesophyll, which increases water vapour pressure in intercellular airspaces (Rockwell *et al.*, 2014; Buckley *et al.*, 2017), favouring stomatal opening and cooling the leaf (Mott and Peak, 2010; Buckley, 2017). In wheat, high temperatures stimulated stomatal opening to cool the leaf when water supply was unlimited (Chavan *et al.*, 2019, 2022). Hence, increased frequency and intensity of extreme heat waves, especially under water limitation, are likely to reduce the yield of C_4_ crops (Lobell *et al.*, 2013; Challinor *et al.*, 2014; Mueller *et al.*, 2016; Potgieter *et al.*, 2016; IPCC, 2021).

Growth under eCO_2_ and WS can change stomatal anatomy and frequency to restrict water loss (Woodward, 1987; Ristic and Cass, 1991; Lawson *et al.*, 2002; Hetherington and Woodward, 2003; Franks and Beerling, 2009; Fujita *et al.*, 2013; Matrosova *et al.*, 2015; Ouyang *et al.*, 2017), leading to changes in stomatal kinetic responses (Lawson and Blatt, 2014). Stomatal aperture and kinetics are sensitive to leaf water status (Buckley, 2005; Lawson and Blatt, 2014), which is influenced by eCO_2_ and WS. Recent reports highlighted the key role of stomatal kinetics in the thermoregulation of grapevine leaves (Faralli *et al.*, 2022). C_4_ crops, known for their fast and efficient stomatal responses, display a faster rate of closing than opening, favouring water retention over heat dissipation or CO_2_ fixation (Israel *et al.*, 2022). Exploring stomatal kinetics, an expanding area of research (Lawson and Blatt, 2014; McAusland *et al.*, 2016; Israel *et al.*, 2022), can provide insight into possible trade-offs between water conservation and leaf thermoregulation (Leakey *et al.*, 2019).

Hence, our overarching objective was to investigate how the imposition of a future climate scenario (eCO_2_×WS×HS) would impact leaf thermoregulation, and the resulting physiological and biomass responses in a key C_4_ crop. We focused on two sorghum lines with contrasting leaf width (LW), selected based on data from a previous experiment which subjected several sorghum lines of varying LW to HS. In sorghum, greater LW leads to increased boundary layer resistance (δ_bl_), reducing the efficiency of gas exchange through the stomata, and reducing leaf evaporative heat loss, further increasing *T*_leaf_ (Pan *et al.*, 2022). Consequently, LW can influence *g*_s_, as wider leaves must open their stomata more to cool, and because LW and stomatal anatomy are related (Al-Salman *et al.*, 2022, Preprint; Pan *et al.*, 2022). Lower *g*_s_, due to a combination of eCO_2_ and WS, compounded by high *T*_leaf_ during a HS, can cause significant damage, especially in lines with wide leaves. Wider leaves are more susceptible to increases in *T*_leaf_ as they experience higher δ_bl_, but also because larger leaves accumulate heat more easily due to increased light interception (Smith, 1978; Beerling *et al.*, 2001). In contrast, higher sink capacity of wide leaf lines may alleviate photosynthetic feedback limitation under stressful conditions, relative to narrow leaf lines (Roitsch, 1999; Hageman and van Volkenburgh, 2021).

In this study, we exposed two glasshouse-grown lines to eCO_2_, WS, and HS treatments in a multifactorial design to test the following hypotheses: (i) eCO_2_ will stimulate photosynthesis and biomass of sorghum under WS; (ii) both eCO_2_ and WS will reduce *g*_s_, resulting in hotter leaves relative to control conditions; (iii) HS will stimulate stomatal opening to promote evaporative cooling and sustain higher rates of photosynthesis; (iv) more open stomata will be concurrent with faster stomatal opening rates to improve thermoregulation under HS; and (v) wider leaves will have higher *g*_s_ during HS, enabling efficient cooling at the expense of water loss.

## Materials and methods

### Plant material

The two lines were selected based on the results of a previous glasshouse experiment conducted between September 2016 and February 2017. That study screened for association between LW and iWUE under different temperatures in 10 sorghum lines including the two here (Al-Salman *et al.*, 2022, Preprint). Those 10 lines were selected randomly from >500 accessions from the Sorghum Conversion Program (SCP) to meet two criteria: low tillering and variation in LW. The SCP is a backcross breeding scheme in which genomic regions conferring early maturity and dwarfing from an elite donor were introgressed into ~800 exotic sorghum accessions representing the breadth of genetic diversity in sorghum (Stephens *et al.*, 1967). The resulting SC lines are closely related to their exotic progenitor line, but differ dramatically in plant height and flowering time due to the presence of elite donor introgressions (~4% of the genome from the recurrent donor) (Thurber *et al.*, 2013; Tao *et al.*, 2020), to form a segregating population (Rosenow *et al.*, 1997). During the LW×temperature experiment, a HS was imposed and those two lines showed contrasting photosynthetic response to that HS. The narrow-leaf line, FF_SC449-14E, maintained photosynthesis during the 5 d heat wave (HS). The wide-leaf line, FF_SC906-14E, experienced a reduction in photosynthesis during the HS. The LW under each growth temperature for those two lines can be seen in the supplementary material of Al-Salman *et al.* (2022, Preprint).

### Plant culture

Seeds were sown on 18 January 2021 (austral summer) into the pots in which they spent the whole experiment. The 7.5 litre cylindrical pots had 40 cm depth to allow development of a deep root system. The soil substrate used was a blend of soil, sand, and organic material such as decomposed bark. The particle size promoted good drainage and aeration, and avoided water pooling around plant roots. A slow-release fertilizer (Osmocote Plus Organic All Purpose) with N:P:K of 13.4:2.6:1.8, and macro- and microelements, was added at the rate of 18.5 g per pot. Fertilizer was mixed throughout the pot depth, leaving higher amounts in the lower half where roots will grow more densely by the end of the experiment. Pots were randomly distributed within the glasshouse and rotated weekly to minimize microclimate effect.

### Experimental design

There were two watering and two [CO_2_] treatments in four adjacent rooms (8 m long×3 m wide×5 m tall) in a naturally lit, controlled-environment greenhouse (Plexiglas Alltop SDP 16; Evonik Performance Materials, Darmstadt, Germany) at the Hawkesbury Institute for the Environment, Western Sydney University, Richmond, New South Wales, Australia (–33.612032, 150.749098). The two [CO_2_] treatments were set at 400 ppm (aCO_2_) and 780 ppm (eCO_2_), with two aCO_2_ and two eCO_2_ chambers. In each chamber, there were 16 pots: eight for each genotype, with four well watered and four water stressed (see below). This resulted in a multifactorial design at *n*=4 for each specific treatment: line×water level×[CO_2_]×HS×4 replicates plants (64 total plants). Temperature was set at 30 °C in all four chambers during the day, and 18 °C during the night. The ~11 °C diurnal variation was maintained in all treatments by heating and cooling throughout the day–night cycle. Actual CO_2_ concentration and temperature conditions are shown in Supplementary Fig. S1. Relative humidity was kept close to 60% in the four glasshouse chambers (Carel Humidisk 65 humidifier). The photosynthetic photon flux density (PPFD) at canopy height (Apogee quantum sensor, USA) varied with prevailing weather conditions but was equivalent across rooms. Daytime maximum PPFD was often ~1300 µmol m^–2^ s^–1^.

### Water stress (WS) application

Pots were first watered excessively in the evening and weighed the following morning. This was done a few days in a row to establish pot weight at 100% field capacity (FC). All the pots were maintained at FC for the first 4 weeks after germination to ensure good plant growth and root establishment before imposing water stress. The difference between the pot weight at FC and pot weight before daily watering represented the amount of water added every evening to maintain 100% FC. Usually, more than this amount was added every evening to compensate for any drainage. After 4 weeks of growth, watering was withheld from half of the pots (WS, water stress treatment), while the other half were watered continuously at FC (WW, well-watered treatment). From week 3, a selection of pots was weighed weekly to record weekly water loss and estimate daily plant transpiration or water loss. After estimating daily water loss (~43 ml d^–1^), a 20% reduction was imposed on the WS plants (~34 ml d^–1^). This started at week 4, after which WW plants were continuously watered through a drip system. WS plants had the drippers taken off and were supplied with 102 ml of water every 3 d only (34 ml d^–1^ for 3 d).

### Heat wave (WS) treatment

The HS treatment was applied during anthesis. While there was some genotypic and within-line variation for flowering date, as expected most flowering occurred when plants were 2.5–3 months old (11–13 weeks after sowing). The HS was applied on the week of 7 April 2021 (12 weeks after sowing). Plants had observable flowers at this point or were in the booting stage (panicle in flag leaf sheath).

The HS lasted 6 d, and was applied to two of the four glasshouse chambers, one 400 ppm room and one 780 ppm room. Night temperatures were raised to 24 °C, followed by two h at 35 °C from 08.00 h, then 6 h at 43°C from 10.00 h to 16.00 h, then back to 35 °C until 18.00 h, and then finally 24 °C again until the next 08.00 h. The plants were allowed to acclimate for 3 d, then sampling occurred during the last 3 d of the HS. The sampling included gas exchange, fluorometry, thermal imagery, and leaf water status. Supplementary Fig. S1 shows the main chamber conditions during the main measurement period.

### Leaf gas exchange

Leaf gas exchange was measured via infrared gas analysis using a Li-6400XT (Licor Biosciences, Lincoln, NE, USA). Measurements were taken between 10.30 h and 15.30 h, during solar mid-day and for the HS treatment when ambient temperatures were 43 °C. The youngest fully expanded leaf (YFEL) was chosen, and the Li-6400XT cuvette was placed on the portion of the leaf that received direct sunlight (the horizontal part as the leaf curves). With some line differences, the YFEL during the HS was the 10th–13th leaf. Humidity inside the cuvette was maintained between 40% and 50%, with saturating photosynthetically active radiation (PAR) of 2000 µmol m^–2^ s^–1^. CO_2_ concentration was maintained under growth conditions (either 400 ppm or 780 ppm). Temperature was regulated by setting the block temperature (*T*_block_) to either 30 °C for plants in control chambers, or 43 °C for plants in HS chambers. The leaf was left to equilibrate under those conditions until steady state was achieved (usually 5–10 min), after which 3–5 measurements were logged. Key parameters extracted were carbon assimilation rate at growth CO_2_ (*A*_growth_), stomatal conductance at growth CO_2_ (*g*_s_), and the ratio of *A*_growth_ to *g*_s_, iWUE. The *C*_i_ was also recorded. This protocol was followed before, during, and after HS measurements always under sunny conditions. We mainly present the gas exchange data taken during HS (but see Supplementary Fig. S2). The quantum yield of photosystem II (ΦPSII) was measured on the same leaf and the same day period using a PAM-2500 chlorophyll fluorometer (Heinz Walz GmbH, Germany).

### Stomatal kinetic responses to light

This was conducted during the HS treatment. Leaves were subjected to 15–20 min of darkness after steady-state gas exchange was measured, then light was increased to 2000 µmol m^–2^ s^–1^ for 5 min, then reduced to 200 µmol m^–2^ s^–1^ for another 5 min. During the 10 min of this protocol, data were logged every 5 s. The *g*_s_ response during transition from low and high light is represented by a Gompertz curve. We calculated the growth rate coefficient of the curve, *k*, estimating the time taken for *g*_s_ to reach steady state using the model below (Winsor, 1932; Vialet-Chabrand *et al.*, 2013; McAusland *et al.*, 2016):


$$\begin{array}{l} g_{s}(t)=\left(g_{max}-g_{0}\right)e^{-e^{\frac{\left(\lambda -t\right)}{k}}}+g_{0} \end{array}$$ (1)

where *g*_s_(*t*) is stomatal conductance at time *t*; *g*_max_ is the steady-state *g*_s_ at the horizontal asymptote at the end of the response curve; *g*_0_ is the conductance at the start of the light change; *k* is the exponential rise or decay constant that describes time taken to achieve steady-state *g*_s_; and λ is a term that describes the time lag in *g*_s_ response after the light change and before the response curve started. For the opening stomatal response from low to high light, *k* was termed *k*_open_, while *k* for the transition to low light (stomatal closure) was termed *k*_*close*_.

### Percentage increase in *g*_s_

We conducted gas exchange measurements using the penultimate YFEL, 1 week before the HS started in order to provide a baseline (Supplementary Fig. S2). The YFEL was kept for the HS treatment time point. We used different leaves for the main measurements (during HS) because leaves were sampled after gas exchange measurements. These data were specifically used to calculate percentage increase in *g*_s_ from before the HS to during the HS (% *g*_s_ HS).

### Saturating *C*_i_ from *A*_n_–*C*_i_ curves

We conducted *A*_n_–*C*_i_ curves on the same leaves as those we used for other gas exchange measurements during HS. A full analysis of the *A*_n_–*C*_i_ curves was beyond the scope of this study, but they enabled us to calculate the difference between saturating *C*_i_ from the *A*_n_–*C*_i_ curves and operational *C*_i_ during the steady-state measurement, termed Δ*C*. This term can highlight whether a leaf is operating at saturating *C*_i_ at steady-state conditions or not (Supplementary Fig. S3).

### Leaf water potential and hydraulic conductance

At the same time of day as the gas exchange measurements, a leaf adjacent to the YFEL used for gas exchange and of similar size was cut, put in a plastic bag with wet tissue, and exhaled into to increase CO_2_ levels. The plastic bag was placed on ice in a dark coolbox. The coolbox was moved to the lab and leaf water potential, Ψ_leaf_, was measured using a Scholander-type pressure bomb (Model 1000 and Model 1505D Pressure Chambers, PMS Instrument Company, Albany, OR, USA). Measurements of Ψ_leaf_ were made a maximum of 3–4 h after cutting and placing in the cool box.

Leaf hydraulic conductance (*K*_leaf_) was estimated by combining measured leaf water potential and leaf energy balance modelling following similar estimates in Robson *et al.* (2012) and Simonin *et al.* (2015). Before sampling for Ψ_leaf_, a leaf below the one used for Ψ_leaf_ was wrapped in plastic and covered with tin foil, and left to acclimate for at least 1 h. This allowed the leaf to equilibrate its water potential with the stem water potential. This was used to estimate stem water potential (Ψ_stem_). To estimate transpiration rate during the time of sampling, the environmental variables at the time of sampling were recorded and leaf energy balance was used to calculate transpiration (*E*). See Supplementary Protocol S1 for full details. *K*_leaf_ was then calculated as:


$$\begin{array}{l} K_{leaf}=\;\frac{E}{(\Psi_{stem}-\;\Psi_{leaf)}} \end{array}$$ (2)

### Thermal imagery

Following gas exchange measurements, we used an infrared thermal imaging camera to measure leaf temperature (T640; FLIR Systems, Wilsonville, OR, USA). A platform was set in the middle of each chamber in an area where sunlight was constant and uniform, and each plant in the room was moved to that position, 1 m away from the camera lens. A thermal image was taken of the upper part of the plant where a YFEL was apparent and was receiving sunlight. The temperature of this leaf was *T*_leaf_. To measure background ambient air temperature, a crumpled tin foil sheet was placed next to the plant and the temperature of the sheet was considered as ambient air temperature, *T*_air_. The difference of *T*_leaf_–*T*_air_, which indicates the capacity of the leaf to cool itself, was Δ*T*.

### Leaf composition and morphology

Leaf discs were taken from the YFEL used for gas exchange using a 0.5 cm^2^ leaf cork borer. FW was first measured, then the leaf discs were submerged in water overnight. The following day, turgid weight (TW) was measured. The leaf discs were then dried at 70 °C for 72 h to obtain the DW. Leaf mass per area (LMA) was calculated as DW divided by the leaf discs’ total area and expressed in g cm^–2^. Relative water content (%, RWC), was calculated as: [(FW–DW)/(TW–DW)]×100. The dried leaf discs were then used to obtain percentage nitrogen content (% N) via a CHN analyzer (LECO TruMac CN-analyser, Leco corporation, USA) using the Dumas dry combustion method. LW was measured on the same leaf, by determining the length of the leaf, and measuring LW at the middle of the leaf.

### Plant characteristics and final harvest

Throughout the experiment, plant height (PH) and leaf number (LN) were recorded every week. Final LN is presented (Supplementary Fig. S4). Plant growth rate (GR) was calculated as the increase in PH per week (cm week^–1^). Flowering date (FD) was recorded as the week in which the flower head was first observed for each plant (outside the flag leaf sheath). At the end of the experiment (~15 weeks after germination), shoot biomass (stem and leaves) and the grain-carrying panicle were harvested, and dried at 70 °C for 72–96 h. The leaves originally sampled for water potential or gas exchange were dried after their area (LA) was scanned. The mass of those leaves was added to the final harvest data. Total shoot vegetative biomass (Biomass) was the total dry weight of the stems and leaves. Panicle size was the dry weight of the grain-carrying heads, and added to the Biomass represents total above-ground biomass.

### Statistical analysis

**Fig. 1.**
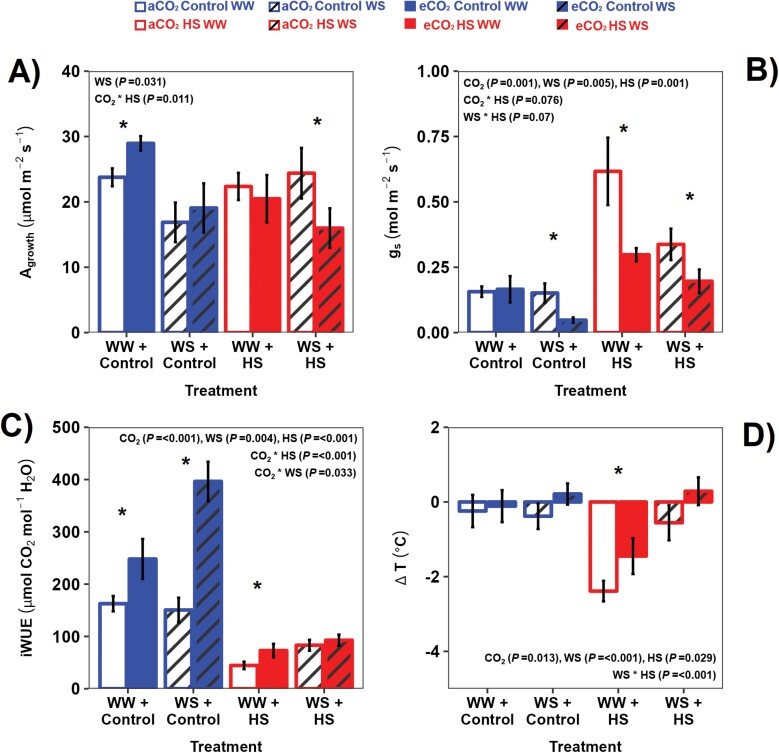
Response of leaf gas exchange and leaf temperature differential to combined treatments of water (well-watered, WW; water stress, WS), [CO_2_] (400 ppm, aCO_2_; 780 ppm, eCO_2_), and extreme temperatures [31 °C, control; 43 °C, heat stress (HS)] in sorghum. See the Materials and Methods for water stress, [CO_2_], and temperature treatment implementation, and for measurement conditions. Gas exchange measurements were made using an infrared gas analyser (Li-6400XT). Leaf and air temperatures were measured using infrared thermography. Each bar chart represents the combined mean of the lines sampled at that treatment combination (*n*=8; error bars=SE). An asterisk represents a statistically significant variation between the two [CO_2_] treatments at *P*<0.05, while statistical information in the insert is the result of a MANOVA (see Table 1). (A) Carbon assimilation rate (*A*_growth_); (B) stomatal conductance (*g*_s_); (C) intrinsic water use efficiency (iWUE); (D) leaf-to-air temperature differential (Δ*T*).

Statistical analysis, data visualization, and model implementation were performed using R (R Core Team, 2020). Normality was checked by plotting a generalized linear model and inspecting residual plots. ANOVA and multiple ANOVA (MANOVA) were carried out using linear mixed-effects models (package nlme), with replicate as the random variable and the fixed variables being [CO_2_]×HS×water treatment×line to obtain the *F*-statistic and *P*-value associated with the model. For statistical difference between [CO_2_] treatments presented with an asterisk in the figues (e.g. Fig. 1), a Student’s *t*-test was carried out between the samples of each specific treatment combination with Bonferroni *P*-adjustment used as multiple comparison correction. For non-normal/parametric data, a Kruskal–Wallis test was conducted. Unless mentioned otherwise, only statistically significant results (*P*<0.05) from the MANOVA and *t*-test comparisons are highlighted in the Results below. Regression analysis was carried out using linear modelling (lm). A Pearson product moment correlation analysis was performed to test statistical significance of relationships and obtain ­correlation coefficients. A spline function was used to separate the segments of the light transient used to estimate stomatal kinetics and, for each segment, the model in Equation 1 was applied. Tables 1 and 2 show all the parameters presented and their abbreviations.

**Table 1.** Summary of *P-*values from the full-factorial mixed effect MANOVA of the parameters

	df	*A*_growth_	*g*_s_	iWUE	*C*_i_	*T*_leaf_	Δ*T*	ΦPSII	Ψ_leaf_	*K*_leaf_	RWC	LMA	% N	*k*_open_	*k*_close_	LW	Panicle	Veg Biom	Tot Biom	Leaf Num	GR	Date
**Line**	1	0.896	0.063	0.095	0.95	0.204	0.768	0.755	0.441	0.654	**0.016**	0.489	**<0.001**	0.89	0.923	**0.044**	0.56	0.514	0.397	0.488	**<0.001**	0.072
**CO**_**2**_	1	0.967	**0.001**	**<0.001**	**<0.001**	**0.006**	**0.013**	0.851	0.706	0.395	0.914	0.441	0.35	0.881	0.123	**0.002**	0.348	**0.009**	**0.039**	0.078	**0.04**	0.905
**Water stress (WS)**	1	**0.031**	**0.005**	**0.004**	0.953	**0.033**	**<0.001**	**<0.001**	**<0.001**	0.741	0.955	0.738	0.225	0.704	0.395	0.604	**0.009**	0.186	**0.024**	0.444	0.111	0.137
**Heat stress (HS)**	1	0.37	**0.001**	**<0.001**	**0.001**	**<0.001**	**0.029**	0.159	0.834	**0.001**	**0.001**	0.849	0.052	0.061	0.431	0.532	0.35	0.308	0.309	0.233	0.408	0.132
**Line×CO**_**2**_	1	0.112	0.087	0.328	0.148	0.424	0.33	0.96	0.152	0.415	**0.037**	0.384	0.36	**0.035**	0.61	0.767	0.443	0.811	0.606	0.998	0.146	0.927
**Line×WS**	1	**0.047**	0.18	0.326	0.358	0.138	0.195	0.237	0.903	0.98	0.674	0.725	0.377	0.173	0.785	0.188	0.789	0.174	0.28	0.53	0.248	0.792
**CO**_**2**_ **×WS**	1	0.346	0.376	**0.033**	0.751	0.692	0.703	0.642	0.972	0.267	0.526	0.433	0.611	0.072	0.778	0.568	0.701	0.963	0.748	0.799	0.804	0.605
**Line×HS**	1	**0.016**	0.899	0.778	0.114	0.79	0.465	0.59	0.868	0.951	0.514	0.629	0.233	0.182	0.743	0.082	**0.044**	0.087	**0.035**	0.064	0.192	0.719
**CO**_**2**_ **×HS**	1	**0.011**	0.076	**<0.001**	0.076	0.186	0.334	0.246	0.748	0.669	0.299	0.56	0.285	0.053	0.61	0.596	0.347	0.617	0.365	0.447	0.834	0.28
**WS×HS**	1	0.133	0.07	0.176	0.116	0.008	**0.001**	0.204	0.174	0.745	0.204	**0.021**	0.964	0.241	0.302	0.625	0.206	0.945	0.452	0.743	0.958	0.154
**Line×CO**_**2**_ **×WS**	1	0.855	0.532	0.156	0.303	0.855	0.084	0.765	0.174	0.337	0.239	0.609	0.329	0.679	0.999	0.171	0.576	0.217	0.24	0.316	0.98	0.676
**Line×CO**_**2**_ **×HS**	1	0.958	0.484	0.103	0.366	0.457	0.069	0.968	0.244	0.363	0.757	**0.044**	0.98	0.588	0.087	0.65	0.988	0.532	0.78	0.821	0.178	0.49
**Line×WS×HS**	1	0.619	0.238	0.335	0.117	0.712	0.577	0.998	0.59	0.829	0.378	0.398	0.834	0.933	0.963	0.421	0.111	0.667	0.204	**0.05**	0.862	0.699
**CO**_**2**_ **×WS×HS**	1	0.394	0.174	**0.005**	**0.002**	0.328	0.181	0.355	0.587	0.215	0.628	0.377	0.094	0.97	0.092	0.062	0.154	**0.021**	**0.03**	0.415	0.325	0.241
**Line×CO**_**2**_ **×WS×HS**	1	0.813	0.247	0.155	0.06	0.493	0.887	0.748	0.277	0.379	**0.029**	0.337	0.354	0.239	0.21	0.831	0.881	0.464	0.634	0.907	0.786	0.59

Abbreviations: *A*_growth_, carbon assimilation rate at growth [CO_2_] (μmol m^–2^ s^–1^); *g*_s_, stomatal conductance at growth [CO_2_] (mol m^–2^ s^–1^); iWUE, instantaneous water use efficiency (μmol CO_2_ mol^–1^ H_2_O); *C*_i_, intercellular [CO_2_] (ppm); *T*_leaf_, leaf temperature (°C); Δ*T*, difference between *T*_leaf_ and air temperature (°C); ΦPSII, quantum efficiency of PSII; Ψ_leaf_, leaf water potential (MPa); *K*_leaf_, leaf hydraulic conductance (mmol m^–2^ s^–1^ MPa^–1^); RWC, relative water content (%); LMA, leaf mass per area (g cm^–2^); % N, nitrogen content as percentage of the leaf mass; *k*_open_, opening rate of stomata during transition from low to high light (min); *k*_close_, closing rate of stomata during transition from high to low light (min); LW, leaf width (cm); Panicle, panicle size (g per plant); Veg Biom, above-ground vegetative biomass (g per plant); Tot Biom, total above-ground biomass (g per plant); Leaf Num, total number of leaves per plant; GR, plant growth rate (cm week^–1^); Date, flowering date (week since sowing).

Bold: *P*<0.05; underlined: 0.05<*P*<0.08.

**Table 2.** Summary of means (±SE) for measured variables.

Line	[CO_2_]	Water	Temp.	A_growth_	g_s_	iWUE	C_i_	T_leaf_	ΔT	ΦPSII	Ψ_leaf_	K_leaf_	RWC	LMA	% N	k_open_	k_close_	LW	Panicle	Veg Biom	Tot Biom	Leaf Num	GR	Date
**FF_SC906-14E**	**400**	**WW**	**Control**	25.59 (1.7)	0.2 (0.02)	138.36 (22.77)	127.33 (39.56)	34.3 (0.52)	-0.07 (0.46)	0.28 (0.02)	1.08 (0.09)	3.75 (1.14)	89.87 (0.62)	30.93 (3.87)	1.67 (0.06)	5.06 (1.84)	1.07 (0.14)	3.74 (0.46)	2.28 (0.9)	8.61 (3.38)	13.76 (3.51)	10.67 (0.29)	9.67 (1.26)	13 (0)
	**780**	**WW**	**Control**	27.67 (1.39)	0.14 (0.04)	226.66 (39.82)	350.6 (66.46)	34.88 (0.67)	-0.23 (0.32)	0.25 (0.02)	1.04 (0.21)	2.22 (0.88)	89.18 (0.76)	39.89 (2.04)	1.79 (0.06)	1.18 (0.24)	0.48 (0.11)	4.73 (0.6)	10.29 (5.73)	20.48 (5.69)	29.22 (9.86)	11.25 (1.7)	13.61 (2.13)	11.25 (1.7)
	**400**	**WW**	**HS**	20.44 (1.7)	0.79 (0.24)	34.22 (9.15)	293.74 (16.01)	39.55 (0.6)	-2.15 (0.32)	0.31 (0.02)	1.23 (0.06)	10.5 (1.11)	84.12 (2.05)	38.15 (2.82)	2.14 (0.06)	0.81 (0.25)	0.86 (0.03)	4.9 (0.48)	8.75 (4.33)	19.97 (4.58)	30.27 (9.92)	11 (1.22)	13.24 (2.04)	12.75 (0.63)
	**780**	**WW**	**HS**	14.87 (5.73)	0.3 (0.04)	54.71 (22.91)	278.41 (157.65)	39.93 (0.44)	-2.08 (0.34)	0.36 (0.01)	1.22 (0.09)	22.85 (9.34)	75.42 (4.08)	36.2 (1.57)	1.96 (0.02)	0.66 (0.23)	0.63 (0.18)	4.48 (0.7)	6.47 (4.31)	17.38 (5.62)	23.85 (9.6)	14.25 (0.85)	11.64 (1.26)	14.25 (0.85)
	**400**	**WS**	**Control**	23.6 (1.22)	0.21 (0.03)	117.59 (18.79)	163.04 (33.57)	33.8 (1.06)	-0.53 (0.71)	0.23 (0.04)	1.43 (0.13)	1.54 (0.14)	94.47 (3.35)	36.28 (1.46)	1.75 (0.18)	2.29 (0.67)	1.3 (0.68)	3.32 (0.34)	1.54 (0.7)	8.29 (2.06)	9.83 (2.74)	11.75 (0.63)	10.36 (0.99)	13.5 (0.29)
	**780**	**WS**	**Control**	19.96 (5.87)	0.04 (0.01)	464.58 (59.1)	90.65 (443.11)	35.95 (0.45)	0.67 (0.4)	0.21 (0.04)	2.04 (0.65)	0.83 (0.43)	85.1 (6.73)	38.06 (4.61)	1.82 (0.1)	1.65 (0.64)	0.42 (0.08)	4.28 (0.33)	1.69 (0.45)	15.11 (2.52)	16.8 (2.73)	12.5 (0.96)	12.36 (1.2)	13.67 (0.29)
	**400**	**WS**	**HS**	24.72 (3.69)	0.34 (0.07)	75.59 (8.91)	216.02 (15.11)	42.43 (0.25)	0.05 (0.16)	0.26 (0.03)	1.53 (0.03)	22.34 (10.17)	79.43 (2.58)	32.52 (2.33)	2.06 (0.07)	0.52 (0.17)	0.47 (0.18)	3.43 (0.4)	1.43 (0.74)	6.63 (0.89)	8.61 (1.6)	11 (0.58)	11.43 (1.69)	13.5 (0.35)
	**780**	**WS**	**HS**	14.35 (3.84)	0.18 (0.05)	82.21 (7.67)	573.17 (14.66)	42.05 (0.25)	0.22 (0.13)	0.24 (0.04)	1.47 (0.08)	12.82 (5.22)	82.75 (2.29)	35.16 (1.94)	2.07 (0.05)	1.24 (0.46)	0.33 (0.03)	4.53 (0.1)	2.65 (0.44)	16.18 (1.3)	18.83 (1.37)	11.75 (0.48)	11.99 (0.99)	13.67 (0.29)
**FF_SC449-14E**	**400**	**WW**	**Control**	22.38 (1.77)	0.13 (0.02)	180.81 (12.24)	66 (17.93)	33.9 (0.67)	-0.35 (0.66)	0.32 (0.03)	1.08 (0.18)	1.28 (0.31)	87.87 (2.38)	33.28 (4.7)	2.08 (0.14)	1.68 (0.31)	0.36 (0.13)	3.44 (0.83)	9.05 (7.96)	12.03 (4.47)	21.07 (10.93)	14 (3.19)	21.02 (5.98)	11.33 (1.26)
	**780**	**WW**	**Control**	30.23 (1.64)	0.19 (0.1)	269.35 (70.4)	279.41 (116.73)	35.18 (0.81)	0 (0.86)	0.28 (0.02)	1 (0.04)	0.97 (0.18)	89.3 (0.76)	31.06 (2.85)	2.35 (0.2)	1.96 (0.53)	1.38 (0.27)	4.73 (0.33)	19.49 (10.43)	27.3 (4.57)	46.79 (12.13)	13.5 (2.87)	25.35 (2.5)	11 (0)
	**400**	**WW**	**HS**	24.28 (3.83)	0.45 (0.04)	54.78 (8.94)	250.66 (19.61)	39.7 (0.61)	-2.63 (0.46)	0.33 (0.02)	1.13 (0.13)	19.66 (2.66)	66.34 (5.61)	34.71 (1.24)	2.47 (0.14)	0.61 (0.09)	1.49 (1.2)	3.11 (0.34)	1.82 (0.83)	11.51 (5.8)	13.32 (6.57)	9.25 (0.63)	17.33 (4.48)	12 (0.87)
	**780**	**WW**	**HS**	26.08 (2.74)	0.29 (0.04)	91.12 (4.94)	549.85 (9.72)	40.78 (1.01)	-0.82 (0.83)	0.37 (0.02)	1.12 (0.13)	12.1 (3.27)	79.36 (5.14)	40.76 (3.56)	2.14 (0.17)	1.29 (0.79)	0.25 (0.03)	3.53 (0.49)	2.17 (1.06)	14.31 (6.75)	16.48 (7.45)	10.5 (0.5)	22.49 (4.76)	12.75 (0.63)
	**400**	**WS**	**Control**	10.14 (3.32)	0.09 (0.05)	183.31 (38.71)	80.13 (57.8)	33.36 (0.57)	-0.24 (0.18)	0.22 (0.04)	1.82 (0.2)	0.84 (0.32)	83.02 (3.45)	40.38 (3.05)	2.57 (0.17)	1.75 (1.19)	1.01 (0.37)	3.7 (0.14)	3.88 (1.07)	20.57 (3.67)	24.45 (4.51)	10 (1.08)	18.38 (2.33)	12.67 (0.29)
	**780**	**WS**	**Control**	18.18 (5.58)	0.05 (0.02)	345.21 (19.68)	180.57 (33.56)	34.98 (0.43)	-0.25 (0.27)	0.21 (0.04)	1.34 (0.21)	0.81 (0.22)	84.29 (4.99)	34.22 (1.06)	2.15 (0.05)	2.32 (1.5)	0.5 (0.16)	3.8 (0.33)	1.62 (0.64)	17.11 (3.36)	21.04 (4.5)	12.25 (1.6)	19.68 (2.67)	12.25 (0.75)
	**400**	**WS**	**HS**	24.07 (7.48)	0.33 (0.11)	90.77 (19.75)	194.86 (24.03)	41.03 (0.9)	-1.03 (0.76)	0.22 (0.06)	1.58 (0.17)	20.24 (11.87)	75.95 (2.73)	31.81 (4.27)	2.57 (0.24)	0.7 (0.04)	0.42 (0.14)	3.02 (0.54)	3.93 (1.35)	8.4 (3.59)	11.73 (4.33)	9.5 (0.87)	11.35 (1.29)	13 (0.5)
	**780**	**WS**	**HS**	17.62 (5.12)	0.22 (0.08)	103.18 (20.09)	536.61 (29.68)	42.03 (0.93)	0.35 (0.79)	0.22 (0.08)	1.39 (0.09)	10.87 (0)	75.99 (3.29)	33.55 (4.43)	2.42 (0.19)	3.75 (1.61)	0.34 (0.08)	4.18 (0.24)	3.9 (1.39)	18.69 (1.96)	22.59 (2.36)	11.5 (0.29)	20.7 (2.96)	12.5 (0.65)

Abbreviations:- A_growth_: carbon assimilation rate at growth [CO_2_] (μmol m^-2^ s^-1^); g_s_: stomatal conductance at growth [CO_2_] (mol m^-2^ s^-1^); iWUE: instantaneous water use efficiency (μmol CO_2_ mol^-1^ H_2_O); C_i_: Intercellular [CO_2_] (ppm); T_leaf_: leaf temperature (°C); ΔT: difference between T_leaf_ and air temperature (°C); ΦPSII: Quantum efficiency of photosystem II; Ψ_leaf_: leaf water potential (MPa); K_leaf_: leaf hydraulic conductance (mmol m^-2^ s^-1^ MPa^-1^); RWC: relative water content (%); LMA: leaf mass per area (g cm^-2^); % N: Nitrogen content as percentage of the leaf mass; k_open_: opening rate of stomata during transition from low to high light (min); k_close_: closing rate of stomata during transition from high to low light (min); LW: leaf width (cm); Panicle: Panicle size (g plant^-1^); Veg Biom: aboveground vegetative biomass (g plant^-1^); Tot Biom: Total aboveground biomass (g plant^-1^); Leaf Num: total number of leaves per plant; GR: plant growth rate (cm week^-1^); Date: flowering date (week since sowing).

"Water" is watering treatment, **WW**: Well watered, **WS**: Water limited; "Temp." is temperature treatment, **Control**: Ambient Temperature (31°C), **HS**: Heat Wave (43°C).

## Results

### Photosynthesis was reduced by WS, while *g*_s_ and iWUE mostly responded to eCO_2_ and HS

We compared the responses of leaf gas exchange to eCO_2_, WS, and HS in two sorghum lines with different LW relative to the control treatment (aCO_2_, WW, and control temperatures). We grouped the results from both sorghum lines to simplify the results, because there was little significant line effect overall, although we found a significant line×HS interaction for *A*_n_ (Tables 1, 2).

In both sorghum lines, WS reduced *A*_growth_ and ΦPSII under control temperature, while eCO_2_ tended to stimulate *A*_growth_ under control temperatures and inhibit it under HS (Fig. 1A;  Tables 1, 2; Supplementary Fig. S5B. *g*_s_ increased under HS and decreased at eCO_2_, especially under WS and HS (Fig. 1B; Tables 1, 2). The stomatal response to HS led to a large increase in *C*_i_ (Supplementary Fig. S5A; Tables 1, 2), and to increased ­evaporative cooling (lower leaf-to-air temperature differential, Δ*T*) under HS (Fig. 1D). eCO_2_ had a small warming effect on *T*_leaf_ (Supplementary Fig. S5C; Tables 1, 2). eCO_2_ enhanced iWUE under control temperature but this response was mild under HS (Fig. 1C; Table 1). Comparison of the leaf gas exchange rates before and after the HS showed the large increase in *g*_s_ under HS, while *A*_growth_ responses remained mild (Supplementary Fig. S4).

**Fig. 2.**
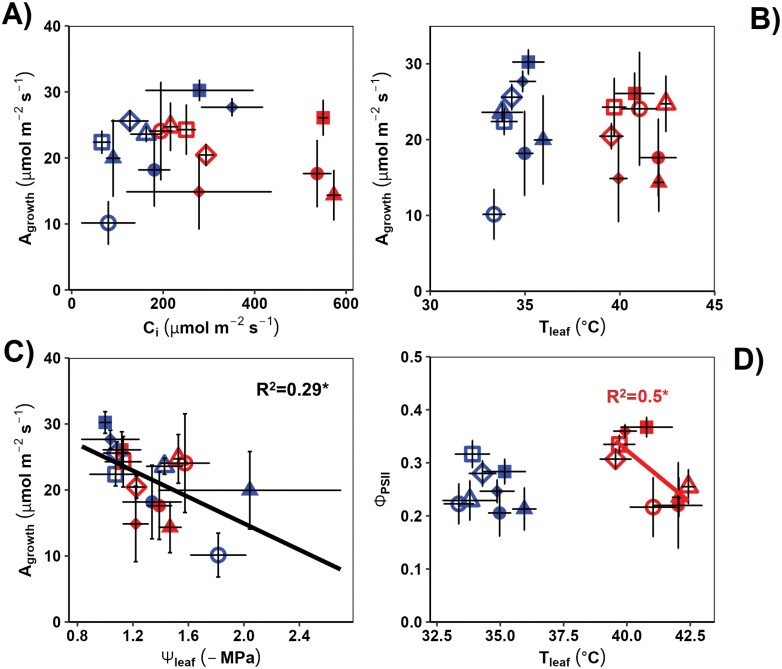
The response of C_4_ photosynthesis in sorghum to leaf physiological stress responses. See the Materials and methods for water stress, [CO_2_], and temperature treatment implementation, and for measurement conditions. Gas exchange measurements were made using an infrared gas analyser (Li-6400XT). Leaf temperature (*T*_leaf_) was measured using infrared thermography. Quantum yield of PSII (ΦPSII) was measured using pulse-amplitude fluorometry. Mid-day leaf water potential (Ψ_leaf_) was measured using a Scholander-type pressure chamber. Solid lines represent the best fit through the data [in D, the red line indicates the fit through heat stress (HS) treatment only]. *R*^2^ values are from a Pearson product–moment correlation analysis (*n*=4; error bars=SE). (A) Carbon assimilation rate (*A*_growth_) versus intercellular [CO_2_] concentration (*C*_i_); (B) *A*_growth_ versus *T*_leaf_; (C) *A*_growth_ versus Ψl_eaf_; (D) ΦPSII versus *T*_leaf_ (control temperatures=blue symbols; HS=red symbols; 400 ppm=open symbols, 780 ppm=filled symbols; well-watered+narrow leaf line= squares; well-watered+wide leaf line=rhombus; water stress+narrow leaf line=circles; water stress+wide leaf line= triangles).

Overall, *A*_growth_ was negatively correlated with leaf water potential (Ψ_leaf_) but was not related to *T*_leaf_ (Fig. 2B, C); however, ΦPSII decreased with *T*_leaf_ under HS (Fig. 2D). *A*_growth_ was generally uncorrelated with *C*_i_, indicating the CO_2_ saturation of C_4_ photosynthesis under most treatments (Fig. 2A). The high *C*_i_ reached under HS was most probably due to the high *g*_s_ rather than metabolic inhibition of photosynthesis, as *A*_growth_ was not much affected by the HS treatment (Fig. 1A).

### Greater evaporative cooling was associated with wider leaves and faster stomatal kinetics

**Fig. 3.**
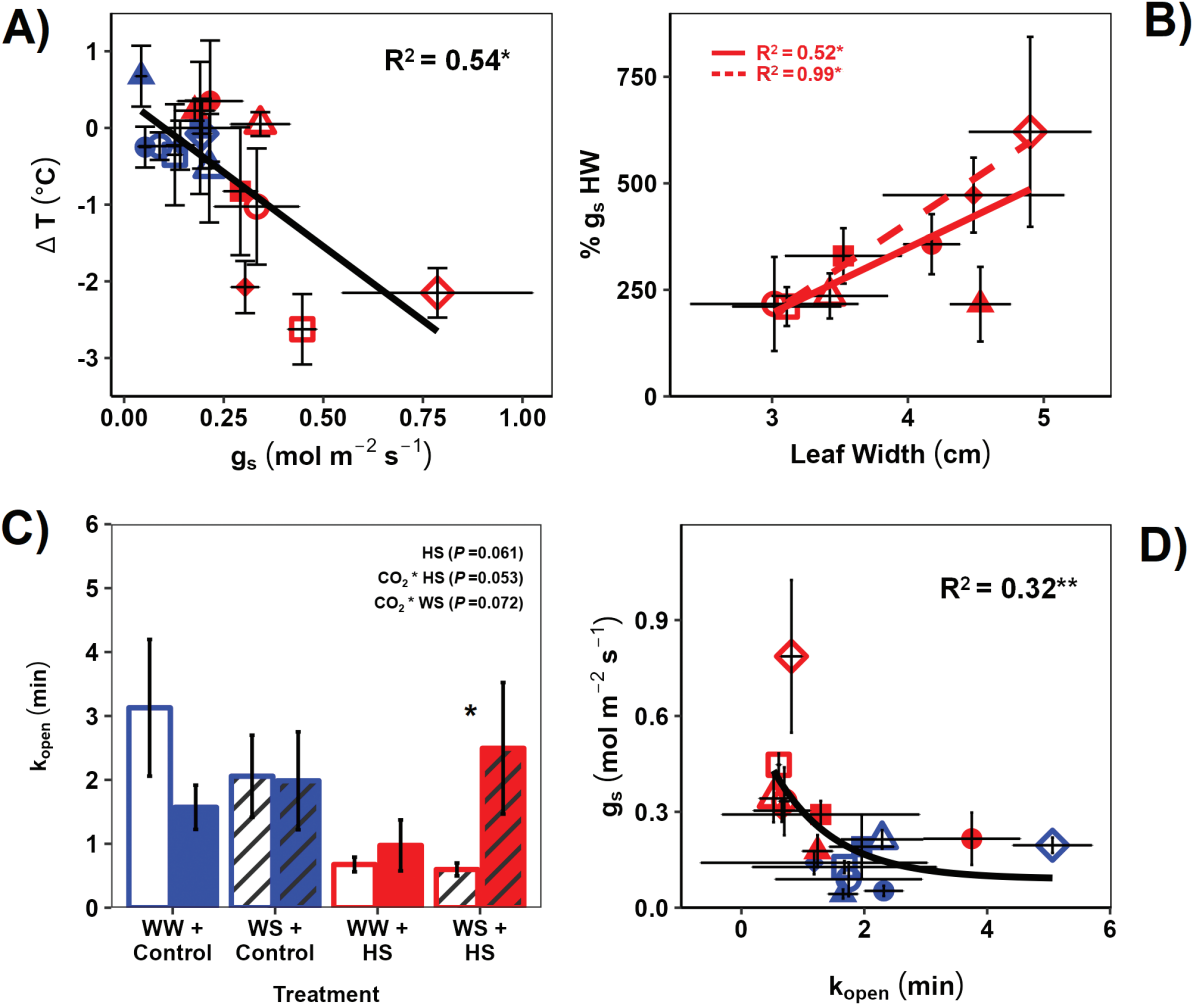
Evaporative cooling by the stomata is linked to leaf width (LW) and stomatal kinetic responses in sorghum. See the Materials and methods for water stress, [CO_2_], and temperature treatment implementation and measurement conditions. (A) Leaf-to-air temperature differential (Δ*T*) versus stomatal conductance (*g*_*s*_); (B) percentage increase in stomatal conductance before to during HS (% *g*_s_ HS) versus LW; (C) response of kinetic constant of stomatal opening during transition from low to high light (*k*_open_) to treatment combinations (see Fig.1 legend for further information); (D) *g*_s_ versus *k*_open_. *R*^2^ in (A) and (B) are from a Pearson product–moment correlation analysis (at *<0.05) (*n*=4). Red fitted line in (B) is the global relationship within the HS treatment, while the dashed line represents the fit through WW plants only. In (C), an asterisk represents statistically significant variation between the two [CO_2_] treatments at *P*<0.05 (*n*=8), while statistical information in the insert is the result of a MANOVA (see Table 1). Each bar chart represents the combined mean of the lines sampled. *R*^2^ in (D) is the adjusted *R*^2^ from an exponential fit (*n*=4) (error bars=SE). See Figs 1 and 2 legends for information and key.

The percentage increase in *g*_s_ during HS relative to the pre-HS values (% *g*_s_ HS) correlated positively with LW, confirming our hypothesis that wide leaves opened their stomata more to achieve efficient cooling (Fig. 3A, B). Consequently, boundary layer conductance (*g*_blw_) was also negatively correlated with Δ*T* (Supplementary Fig. S5E).

The time-constant of stomatal opening (*k*_open_) in response to light transients was lower under HS (*P*=0.061), especially at aCO_2_ (Fig. 3C; Tables 1, 2), and correlated significantly and negatively with steady-state *g*_s_ (slower stomatal opening=lower *g*_s_) (Fig. 3D). *k*_open_ correlated positively with Δ*T* (Supplementary Fig. S5D; adjusted *R*^2^=0.36, *P*<0.05). The time-constant of closing, *k*_close_, displayed less significant relationships but was generally associated positively with higher *g*_s_ (Supplementary Fig. S5F).

### Biomass was stimulated by eCO_2_ under WW conditions

**Fig. 4.**
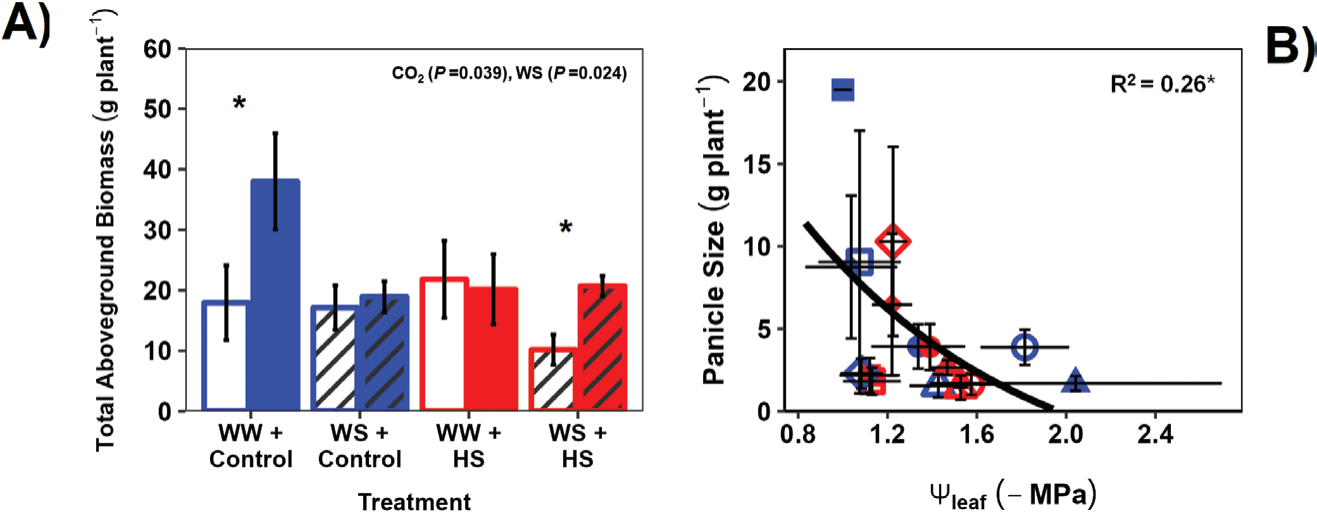
Response of plant biomass and panicle size to combined treatments of water (well-watered, WW; water stress, WS), [CO_2_] (400 ppm, aCO_2_; 780 ppm, eCO_2_), and extreme temperatures [31 °C, control; 43 °C: heat stress (HS)] in sorghum. See the Materials and methods for water stress, [CO_2_], and temperature treatment implementation. In (A), an asterisk represents statistically significant variation between the two [CO_2_] treatments at *P*<0.05 (*n*=8), while statistical information in the insert is the result of a MANOVA (see Table 1). Each bar chart represents the combined mean of the lines sampled. *R*^2^ in (B) is from a Pearson product–moment correlation analysis (at *P<0.05) (*n*=4). (A) Response of total above-ground biomass (vegetative and panicle mass) to the treatments; (B) panicle size versus mid-day leaf water potential (Ψ_leaf_) (error bars=SE). See Figs 1 and 2 legends for information and key.

Total above-ground biomass (vegetative tissues and panicles) was enhanced by eCO_2_ under control temperature and WW conditions (Fig. 4A). Under WS or HS conditions, eCO_2_ did not result in biomass stimulation relative to the control treatment, but eCO_2_ prevented the biomass reduction observed under combined WS and HS at aCO_2_ (Fig. 4A). WS reduced above-ground biomass at eCO_2_ under control temperature and at aCO_2_ under HS (Fig. 4A). Overall, panicle size decreased with increasing Ψ_leaf_ (Fig. 4B; *R*= –0.51, *P*<0.05), highlighting the sensitivity of grain filling to plant water status.

## Discussion

To predict future crop performance and yields, we must consider responses to combined environmental stresses (Watson-Lazowski and Ghannoum, 2021). We used two sorghum lines with contrasting LW and photosynthetic response to temperature to test the interactive impact of future climate conditions (eCO_2_, WS, and HS) on leaf gas exchange, thermoregulation, and plant biomass. The main findings were: (i) eCO_2_ had a positive effect on photosynthesis and biomass under non-stressful conditions; (ii) eCO_2_ had little to negative effects on photosynthesis under the combination of WS and HS; but (iii) eCO_2_ was positive for biomass production under those stresses; and (iv) HS increased *g*_s_ especially in wider leaves and reduced *k*_open_ at aCO_2_ but not at eCO_2_.

### eCO_2_ stimulated photosynthesis and biomass of the C_4_ crop sorghum under non-stressful conditions

Due to the CCM, C_4_ photosynthesis is generally very close to CO_2_ saturation at aCO_2_ (Leakey, 2009), and C_4_ crops are not expected to benefit from increases in atmospheric [CO_2_] under non-limiting water supply (Samarakoon and Gifford, 1996; Ghannoum *et al.*, 2000; Wall *et al.*, 2001). For the two sorghum lines, eCO_2_ enhanced photosynthesis (*A*_growth_) under control water and temperature treatments (Fig. 1A; Supplementary Fig. S2), mainly due to the significant increase in *C*_i_ (Supplementary Fig. S5A). Under control conditions, photosynthesis in both lines was operating below the saturating *C*_i_ as determined by the *A*_n_–*C*_i_ curves (Supplementary Fig. S3). Hence, even in C_4_ sorghum, stomata can impose a limitation on photosynthesis by reducing *C*_i_ below saturation levels, and eCO_2_ can overcome this limitation (Ziska and Bunce, 1997). In fact, the study of the diffusive and biochemical relative limitations to net photosynthesis in several C_4_ grasses revealed that photosynthesis in sorghum was 24, 5, and 4% limited by the stomata, mesophyll, and CO_2_ hydration, respectively, and only 9% limited by phosphoenolpyruvate (PEP) carboxylation (Cano *et al.*, 2019). Hence, under elevated CO_2_, photosynthesis increased because there was higher supply of CO_2_ to the first site of carboxylation, which can fuel Rubisco carboxylation in sorghum, which is higher compared with other C_4_ grasses (Cano *et al.*, 2019). This particular physiology of sorghum (at least within studied varieties) makes it more responsive than other C_4_ grasses to eCO_2_ under WW conditions (Bellasio *et al.*, 2018; Cano *et al.*, 2019). On the other hand, stomatal limitation of C_4_ photosynthesis is observed under WS conditions (Ripley *et al.*, 2007; Ghannoum, 2009). Notwithstanding, several studies reported mild to significant stimulation of photosynthesis in response to eCO_2_ under WW conditions for C_4_ crops and wild C_4_ grasses (Knapp *et al.*, 1993; Wand *et al.*, 1999; Ziska *et al.*, 1999; Cao *et al.*, 2022).

Moreover, the small increase in *T*_leaf_ at eCO_2_ (Supplementary Fig. S5B; Table 1) would have contributed to the increase in *A*_growth_ under WW and control temperatures, due to the leaf approaching its optimum for photosynthesis (±36 °C) (Ghannoum *et al.*, 2000; Siebke *et al.*, 2002; Prasad *et al.*, 2009; Djanaguiraman *et al.*, 2014; Sonawane *et al.*, 2017). Higher temperatures also promote cell expansion and division in C_4_ crops, leading to increased leaf appearance, elongation, and expansion rates (Ben-Haj-Salah and Tardieu, 1995; Granier and Tardieu, 1998; Lafarge *et al.*, 1998; Reymond *et al.*, 2003; Wang *et al.*, 2017). This partially explains the increase in LW and leaf biomass at eCO_2_ (Tables 1, 2). In addition to relieving the diffusional limitation, eCO_2_ reduces leaf transpiration rates, which can improve plant water status and promote greater turgor potential (Wullschleger *et al.*, 2002). Both factors can enhance iWUE under eCO_2_ and promote leaf expansion (as estimated by LW), leaf number, and plant growth rate under eCO_2_ (Supplementary Fig. S4; Tables 1, 2) (Ribeiro *et al.*, 2021; Martínez-Goñi *et al.*, 2022) independently of photosynthesis (Wand *et al.*, 1999; Ghannoum *et al.*, 2000). For example, reduced evaporative demand, such as that elicited by eCO_2_ as it closes the stomata, increased the leaf elongation rate in maize (Ben-Haj-Salah and Tardieu, 1996). Consequently, both increases in *A*_growth_ and *T*_leaf_ by unit of leaf area, leaf expansion rates, and leaf number (due to increased *T*_leaf_ as well as improved water relations) jointly explained the increased biomass by eCO_2_ observed in the two sorghum lines under control conditions (Fig. 4A). Other studies have also reported growth stimulation by eCO_2_ in sorghum under WW conditions (Prior *et al.*, 2003; Wu *et al.*, 2009; Asadi and Eshghizadeh, 2021), and suggest improved water relations, higher *T*_leaf_, and increased *C*_i_ as drivers of biomass accumulation (Asadi and Eshghizadeh, 2021). Nevertheless, we found significant line×CO_2_ interactions for relative water content (RWC) and growth rate (GR) (Table 1), suggesting that growth response to eCO_2_ differs between sorghum genotypes and should be further investigated, especially in conjunction with other stresses such as water or nitrogen limitation (Ribeiro *et al.*, 2021).

### Reduced *g*_s_ and leaf thermoregulation may have restricted the photosynthetic response to eCO_2_ under combined WS and HS

Under aCO_2_ and WW conditions, higher temperatures during HS encourage latent heat transfer from the mesophyll to the intercellular airspaces, increasing vapour pressure in the substomatal cavity, which opens the stomata and propels evaporative cooling (Fig. 3A) (Shope *et al.*, 2008; Mott and Peak, 2010; Pan *et al.*, 2022). This mechanism, which controls leaf energy balance (Buckley *et al.*, 2017), was impacted by lower *g*_s_ under eCO_2_ and WS (Fig. 3A, B). This reduces the rate of latent heat transfer and consequently water vapour diffusion to ambient air (Rockwell *et al.*, 2014). Consequently, leaves heat up more easily under eCO_2_ and WS. Hence, the widely observed reduced *g*_s_ response under eCO_2_ (Morison, 1998), including in C_4_ plants (Knapp *et al.*, 1993; Wand *et al.*, 1999, 2001; Taylor *et al.*, 2018), serves to conserve water when CO_2_ is abundant at the expense of higher *T*_leaf_ (Fig. 3; Supplementary Fig. S5). The impact of reduced *g*_s_ at eCO_2_ on leaf temperature in C_4_ species has been previously established (Thurman and Martin, 2000; Siebke *et al.*, 2002). Here, we also show that even during a 6 d HS, where *g*_s_ doubled to cool the leaf, eCO_2_ further reduced *g*_s_ compared with aCO_2_, increasing Δ*T* (Figs 1, 3A).

We hypothesized that LW will affect leaf thermoregulation. Wider leaves have thick boundary layers, which led, especially under low wind speed, to increased diffusive resistance to transpiration, restricting convective heat transfer (Gates, 1968; Nobel, 2009; Monteith and Unsworth, 2013; Pan *et al.*, 2022). Hence, wider leaves are likely to get hotter unless they significantly increase their *g*_s_ to facilitate transpirational cooling. Under HS, wide leaves significantly increased their *g*_s_ more than narrow leaves, especially when water for transpiration was not limiting (Figs 1D, 3B). The increase in LW under eCO_2_ may have dampened leaf thermoregulation, exacerbating the impact of future heat waves.

Despite higher *g*_s_ and better thermoregulation under HS (Fig. 1B, D), eCO_2_ caused a photosynthetic decrease under combined stresses (HS and WS), especially in the wider leaf line (Fig. 1A; Table 2). *C*_i_ was very high under this treatment combination (eCO_2_× HS×WS), indicating that reduced photosynthesis was due to metabolic inhibition, possibly due to supra-optimal *T*_leaf_, with eCO_2_×WS×HS having the highest mean *T*_leaf_ of all treatments (Table 2; Supplementary Fig. S5C). While there was no correlation between *A*_growth_ and *T*_leaf_ considering all the treatments (Fig. 2B), ΦPSII and *T*_leaf_ correlated negatively during HS (Fig. 2D), and both *A*_growth_ and ΦPSII were lower under WS (Table 1). The C_4_ photosynthetic apparatus is sensitive to WS, and can be inhibited by biochemical, non-stomatal factors that are sensitive to high temperatures (Ghannoum *et al.*, 2003; Ghannoum, 2009). Soil water deficit can inhibit the electron transport chain and the Calvin cycle activity, reducing electron drawdown from the photosystems. Both responses lead to build up of excess light energy under high light, causing photodamage (Osmond and Grace, 1995; Cornic and Fresneau, 2002; Cousins *et al.*, 2002). Hence, it is possible that the main impact of combined WS and eCO_2_ under HS was related to high *T*_leaf_ that caused biochemical and photochemical inhibition.

### Stomata opened faster during extreme heat under aCO_2_ but not eCO_2_

eCO_2_, WS, and LW can affect stomatal anatomy and hence kinetics (Drake *et al.*, 2013; Digrado *et al.*, 2022; Israel *et al.*, 2022; Pan *et al.*, 2022). Faster stomatal opening during HS (i.e. HS decreased *k*_open_) led to considerably cooler leaves (Fig. 3C; Supplementary Fig. S5D), because they maintained higher *g*_s_ and transpirational cooling (Fig. 3A, D), as was recently observed in grapevine leaves (Faralli *et al.*, 2022). The temperature responses of stomata are still not fully elucidated (Mott and Peak, 2010), but it is likely that the main impact of temperature would be in changing evaporation rates and vapour pressure gradients inside the leaf (Buckley *et al.*, 2017; Buckley, 2019). The reduction in viscosity of water under high temperature along with the temperature dependence of outside-xylem water flow probably led to increased hydraulic conductivity, *K*_leaf_, as observed here under HS (Tables 1, 2). The increase in *K*_leaf_ under HS can explain the decrease in *k*_open_ despite the increase in ambient VPD (Mott and Peak, 2010; Buckley, 2019). Additionally, high temperatures have been shown to influence potassium ion channel conductance in *Vicia faba*, maintaining open stomatal pores (Ilan *et al.*, 1995).

Growth under eCO_2_ significantly restrained the fast-opening response under HS, especially during WS (Fig. 3C), leading to hotter leaves. Slower stomatal opening and faster closing (Supplementary Fig. S5F) are water conservation responses, and past studies have shown a convergence between the ­stomatal response to CO_2_ and the WS-induced hormone abscisic acid (Raschke, 1975; Engineer *et al.*, 2016). This convergence can explain why the eCO_2_ effect on *k*_open_ was more prominent in the WS and HS treatment (Fig. 3C). A possible cause is that under eCO_2_ there was a higher CO_2_ concentration around the guard cells that promoted the tendency to close the stomata (Takahashi *et al.*, 2022). Moreover, eCO_2_ reduces stomatal density and increases stomatal size (Franks and Beerling, 2009; Taylor *et al.*, 2012). This anatomical combination has been associated with slower stomata in a range of species (Drake *et al.*, 2013; Lawson and Blatt, 2014; Raven, 2014). eCO_2_ can increase leaf size, as it did here (Tables 1, 2), which exacerbates the low density–larger size stomatal trade-off (Baird *et al.*, 2021; Al-Salman *et al.*, 2022, Preprint; Pan *et al.*, 2022).

### eCO_2_ prevented reduction of biomass during HS

The combination of WS and HS reduced above-ground biomass of the sorghum plants under aCO_2_ but not under eCO_2_ (Fig. 4A). This is an important observation as it means there may be increased crop yield stability under a future eCO_2_ world in the face of climatic extremes (Powell *et al.*, 2012; Ainsworth and Long, 2021; Abdelhakim *et al.*, 2022). Several studies found that growth under eCO_2_ maintained or stimulated shoot biomass as well as photosynthesis and iWUE under WS in sorghum (Ottman *et al.*, 2001; Asadi and Eshghizadeh, 2021; Martínez-Goñi *et al.*, 2022). In our case, biomass maintenance at eCO_2_ under WS and HS came despite photosynthetic reduction during the 6 d of HS (as highlighted above). Therefore, eCO_2_ indirectly alleviated plant growth under combined stresses, most probably due to the benefits of reduced *g*_s_ on plant and soil water status upon imposition of WS (i.e. 7–8 weeks before HS). At aCO_2_, HS greatly increased *g*_s_ which promoted transpiration, reducing soil water content, and acting like additional WS for the plants (Fig. 1). The increase in *g*_s_ was curbed by eCO_2_, reducing whole-plant transpiration, and possibly keeping the soil wetter for prolonged leaf growth and photosynthesis during WS (Seneweera *et al.*, 2001). This is particularly relevant as both sorghum lines are ‘stay-green’ (Borrell *et al.*, 1999). Lower transpiration may have also improved plant water status, as Ψ_leaf_ of the HS×WS treatment was lower at aCO_2_ compared with eCO_2_ (Table 2), similar to findings in Martínez-Goñi *et al.* (2022). RWC was affected negatively by HS (Tables 1, 2), which would have exacerbated the WS effect, making the eCO_2_ effect on leaf water status more conspicuous at HS×WS compared with HS×WW. Hence, it is likely that the mitigating effect of eCO_2_ on plant biomass was mediated by stomatal responses, because although the line with wider leaves showed lower *A*_growth_ during HS (Tables 1, 2), generally photosynthesis was not inhibited by HS in sorghum (Fig. 1), as also found in wheat (Chavan *et al.*, 2019).

Finally, sink variation can be an important determinant of photosynthetic changes during the transfer from vegetative to reproductive phase (Dingkuhn *et al.*, 2020). Both lines were into the reproductive phase during the HS. High air temperatures have been shown to impact yield and cause sterility in graminoid crops (Matsui *et al.*, 1997; Prasad and Djanaguiraman, 2011), which can be alleviated by evaporative cooling (Julia and Dingkuhn, 2013; Prasad *et al.*, 2019). Hence, it is possible that the reduction in photosynthesis observed under WS×HS×eCO_2_ treatment might also be due to sink limitation under the combined stresses. The correlation between panicle size and Ψ_leaf_ (Fig. 4B) is not conclusive but points to a relationship in this direction. The less stressed (and less hot) the leaves, the better the yield.

### Conclusion

We grew two sorghum lines in a multifactorial design to test the impact of future climate conditions on leaf thermoregulation, photosynthesis, stomatal kinetics, and above-ground biomass. eCO_2_ stimulated photosynthesis under non-stressful conditions by overcoming diffusional limitations. Under WS and HS, however, this advantage was lost because of biochemical or photochemical damage caused by high *T*_leaf_. HS increased *g*_s_, particularly in wider leaves, and reduced *k*_open_ at aCO_2_ but not at eCO_2._ Overall, eCO_2_ increased plant biomass, even when photosynthesis was reduced during HS. Taking into account the limited number of germplasms and replications, and the complex environmental combinations, our results point to the interactive effects of eCO_2_ on the contrasting roles of stomatal-driven water conservation associated with drought tolerance in sorghum (low *g*_s_ and water saving) and stomatal-driven leaf thermoregulation (high *g*_s_ and photosynthesis) under water and heat stress.

## Supplementary data

The following supplementary data are available at *JXB* online.

Protocol S1. Calculations of leaf hydraulic and boundary layer conductance.

Table S1. MANOVA results of the main parameters measured before and after the HS.

Fig. S1. A snapshot of the environmental conditions in the glasshouse chambers during the measurement period.

Fig. S2. Comparison of gas exchange variables before, during, and after the HS treatment.

Fig. S3. Response of saturating *C*_i_ to operating *C*_i_ differential to the treatments.

Fig. S4. Response of leaf number and growth rate to the treatments.

Fig. S5. Response of intercellular [CO_2_], leaf temperature, and stomatal kinetics to the treatments, and correlations between them.

Fig. S6. Relationship between total leaf area and total above-ground biomass.

erad063_suppl_Supplementary_Material

## Data Availability

The data generated and analysed for this study are available from the corresponding author on request.
