# Supplementary material for: Elevated [CO2] negatively impacts C4 photosynthesis under heat and water stress without penalizing biomass
Source: J Exp Bot. 2023 Feb 17;74(9):2875–90. doi: 10.1093/jxb/erad063 (PMC10401618; doi:10.1093/jxb/erad063)
Supplement: erad063_suppl_Supplementary_Material [file erad063_suppl_supplementary_material.pdf]

Protocol S1

We used energy balance modelling to calculate hydraulic conductivity and boundary layer conductance, as well as estimating other measures of leaf temperature.

First we need to calculate the saturation water vapour pressure within the leaf $e\left(T_{leaf}\right)\ in\ kPa$, from leaf temperature (T_{leaf} in ºC) following (Buck, 1981):

$$e_{ias}\left(T_{leaf}\right) = 0.61365e^{\frac{17.502T_{leaf}}{240.97+T_{leaf}}} \qquad eq.(S1)$$

Where

$$T_{leaf}(ºC) = \begin{cases} T_{air}, & g_{sw} \geq 0.2\ mol\ H_2O\ m^{-2}s^{-1} \\ T_{air} - 10g_{sw} + 2, & g_{sw} < 0.2\ mol\ H_2O\ m^{-2}s^{-1} \end{cases}$$

T_{air} is the ambient temperature inside the glasshouse chamber at the time of measurement/sampling.

The saturation water vapour pressure in the air surrounded by the leaf:

$$e_{air}(T_{air}) = 0.61365e^{\frac{17.502T_{air}}{240.97+T_{air}}} \qquad eq.(S2)$$

The air around the leaf is not saturated in water vapour, hence the water vapour pressure in the air surrounded by the leaf would be the:

$$e_{air}(T_{air})\frac{RH}{100} \qquad eq.(S3)$$

where, RH is the air relative humidity in percent (%). Now, we can calculate the Vapor Pressure Deficit (VPD, kPa). This is the difference in vapor pressure between the substomatal cavities and what is actually in the air.

$$VPD = e_{ias}\left(T_{leaf}\right) - e_{air}(T_{air})\frac{RH}{100} \qquad eq.(S4)$$

The VPD (kPa) would be the “driving force”, however, is not technically a force, as it does not have the proper units for force, that allow a flux density or transpiration rate (E in mol H_2O m^{-2} s^{-1}) for a given water vapour conductance from the intercellular air spaces to the surrounded air (g_{tw}) divided by the atmospheric pressure in kPa by:

$$E(mol\ H_2O\ m^{-2}s^{-1}) = VPD(kPa)\frac{g_{tw}(mol\ H_2O\ m^{-2}s^{-1})}{P(kPa)} \qquad eq.(S5)$$

The total leaf conductance to water vapour (g_{tw}), which includes stomatal and boundary layer conductances, expressed in mol H_2O m^{-2} s^{-1}, is calculated from the measured stomatal conductance to water vapour (g_{sw}, mol H_2O m^{-2} s^{-1}) at the same PPFD than in ambient light. We measured PPFD during the time of sampling using a light meter and estimated gs by finding the gs at those light levels from light response (A-Q) curves conducted during the experiment. The effective boundary layer conductance to water vapour (g_{blw_effect}) acting in series:

$$g_{tw} = \frac{1}{\frac{1}{g_{sw}} + \frac{1}{g_{blw\_effect}}} \qquad eq.(S6)$$

The effective boundary layer conductance to water vapour for the leaf and considering both sides of the leaf (g_{blw_effect}) should be computed as:

$$g_{blw\_effect} = \frac{g_{blw}}{\frac{Stoma_{ratio}{}^2 + 1}{(Stoma_{ratio} + 1)^2}} = \frac{g_{blw}}{k_f} \qquad eq.(S7)$$

Where the stomatal ratio ($stoma_{ratio}$) may be approximated to the ratio of the stomatal density in the adaxial side to the abaxial one. However we may assume a stomatal ratio of 1 by default (as in the gas *exchange* calculations), what means equal stomatal density in both leaf sides. The resultant k_f is 0.5 and g_{blw_effect} equal $2g_{blw}$. If the averaged stomatal ratio were about 0.7 (than in other sorghum studies, e.g. Pan et al. 2021) then k_f = 0.5156 and hence there would be not big difference when calculating the boundary layer conductance if assumed stomatal ratio of 1 or to include the individual value for each genotype.

We calculated the one-sided leaf boundary layer conductance for water vapour diffusing across the boundary layer of air next to leaf surface (g_{blw}) from the one-dimensional form of Fick's first law of diffusion, as defined in :

$$g_{blw}(mol\ H_2O\ m^{-2}s^{-1}) = \frac{D_{air-H2O}(\mathrm{m^2\ s^{-1}})}{\delta_{bl}(m)} \frac{P(Pa)}{R(m^3\ Pa\ mol^{-1}\ K^{-1})T_{leaf}(K)} \qquad eq.(S8)$$

Where, P represents the atmospheric pressure in **Pa** (note that in equation 5 the pressure P is given kPa as in the licor File , e.g. Press = 101 kPa, but here P = 101000

Pa), T_{leaf} in Kelvin degrees (i.e. $T_{leaf}\,(K) = T_{leaf}(ºC) + 273.15$) and R the gas constant (equal to 8.314 m^3 Pa mol^{-1} K^{-1}).

$D_{air-H2O}$ is the diffusion coefficient for water vapor in air corrected by temperature and pressure followed the equation described by (Marrero and Mason, 1972) and modified to have units of m^2 s^{-1}:

$$D_{air-H2O} = \frac{e^{\left(Ln\,(A)+sLn(T_{leaf})-\left(\frac{S}{T_{leaf}}\right)\right)}}{10000}\frac{101325}{P(Pa)} \qquad eq.(S9)$$

where "A", "s" and "S" are empirical constants with values for the diffusion of water vapour in air of "A" = 0.00000187 (atm cm^2 s^{-1} $(K)^{-s}$); "s" = 2.072; and "S" = 0 (K) and "T_{leaf}" is the temperature in Kelvin degrees (K) and Ln is the natural logarithm. For dilute gases, like water vapour in air, the diffusion coefficient is inversely proportional to pressure, thus $D_{air-H2O}$ was further corrected by atmospheric pressure using 1 atm = 101325 Pa and P the pressure at the glasshouse measured by licor e.g. = 101000 Pa.

and δ_{bl} is the average boundary layer thickness of one side of a leaf, estimated following (Nobel, 2009):

$$\delta_{bl}(m) = \frac{d}{1000}\sqrt{\frac{l(m)}{v(m\,s^{-1})}} \qquad eq.(S10)$$

Where *l* (m) is the mean length of the leaf in the downwind direction in meters, which in grasses matches with the leaf width, and *v* (m s^{-1}) is the wind speed of air inside the glasshouse in m s^{-1}, assumed to be low from personal appreciation e.g. 0.3 m s^{-1}. The increase of 3% of δ_{bl} for every increase of 10 ºC of air temperature above 20 ºC (Schlichting and Gersten, 2016), is incorporated in the formula through the coefficient d:

$$d = 3.76 + 0.012T_{leaf} \qquad eq.(S11)$$

Darcy's law can be expressed in terms of hydraulic conductance between two points in the flow (Sperry *et al.*, 1998; Robson *et al.*, 2012) what allowed to calculate the leaf hydraulic conductance (k_{leaf}) and the whole plant hydraulic conductance (k_{plant}) by:

$$k_{leaf}(mmol\ H_2O\ m^{-2}s^{-1}MPa^{-1}) = \frac{E}{\Delta\psi} = \frac{E\ (mmol\ H_2O\ m^{-2}s^{-1})}{(\psi_{leaf} - \psi_{stem})(MPa)} \qquad eq.(S12)$$

Where ψ_{leaf} is the leaf water potential measured by pressure bomb at midday in transpiring leaf, ψ_{stem} is the water potential in the stem measured in a non-transpiring leaf opposite to the transpiring leaf. And *E* is the transpiration rate calculated by equation 5 and expressed in mmol H_2O m^{-2} s^{-1}.

Table S1. Summary of *F*-Statistic and significance levels from a multi-factorial ANOVA on parameters sampled before and after the heat stress timepoint. (**: *P*<0.0001; *:*P*<0.05; underlined: *P*<0.08).

			Before			After		
**Line**	**Heat Treatment**	**Parameter**	**CO_2**	**Water**	**CO_2 x Water**	**CO_2**	**Water**	**CO_2 x Water**
**FF_SC906-14E**	**Control Plants**	A_{growth}	0.74	0.06	1.77	**5.57***	2.23	**6.28***
		g_s	**10.05****	0.71	1.03	**24.48***	1.37	0.03
		iWUE	**39.33***	0.19	0.02	**77.22***	1.65	0.41
		C_i	**12.97***	0.37	3.07	3.73	1.5	**21.85***
		LW	**7.46***	0.73	0.42	**12.99***	**8.19***	0.01
	**Heat Wave Plants**	A_{growth}	0.98	0.05	2.52	0.19	2.01	0.01
		g_s	**17.67***	0.02	0.9	2.68	1.79	0.09
		iWUE	**48.48****	0.14	0.01	**27.75****	2.15	1.25
		C_i	2.96	0.08	0.01	0.06	0.76	1.84
		LW	1.18	1.42	3.73	0.43	4.09	2.13
**FF_SC449-14E (Narrow-leaf)**	**Control Plants**	A_{growth}	**8.53***	**16.43***	0.68	0.74	**5.19***	0.4
		g_s	0.35	**9.9***	0.31	**26.49****	**13.3***	3.9
		iWUE	**35.4***	0.18	0.37	**26.23****	0.77	0.047
		C_i	<u>4.48</u>	0.08	0.69	**7.34***	0.25	0.01
		LW	2.34	0.05	1.55	1.59	2.79	3.63
	**Heat Wave Plants**	A_{growth}	0.04	<u>4.47</u>	0.004	0.23	**13.62***	0.11
		g_s	**19.59***	**7.19***	0.35	**8.00***	2.66	0.21
		iWUE	**160.7****	**9.43***	5.64**	**17.45***	3.34	0.03
		C_i	0.42	**6.23***	<u>4.76</u>	**7.95***	4.52	0.09
		LW	3.2	1.1	0.44	2.27	0.01	1.04

Abbreviations:- A_{growth}: carbon assimilation rate at growth [CO_2] (μmol m^{-2} s^{-1}); g_s: stomatal conductance at growth [CO_2] (mol m^{-2} s^{-1}); *iWUE*: instantaneous water use efficiency (μmol CO_2 mol^{-1} H_2O); C_i: Intercellular [CO_2] (ppm); *LW*: leaf width (cm).

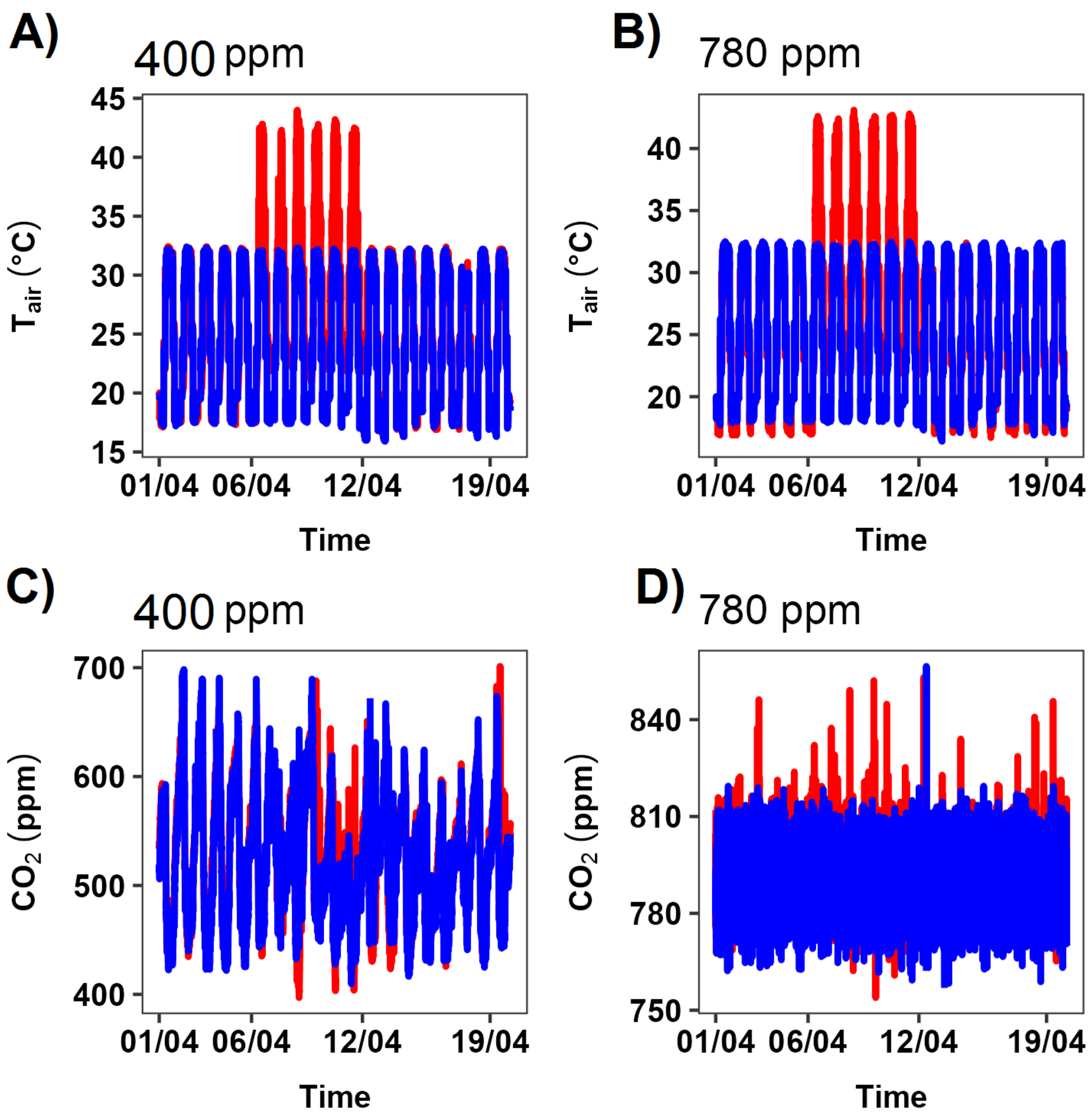

Fig.S1 A snapshot of the environmental conditions in the glasshouse chambers during the main measurement period. The blue line represents the control chamber, the red line the chamber that experienced a heat wave.

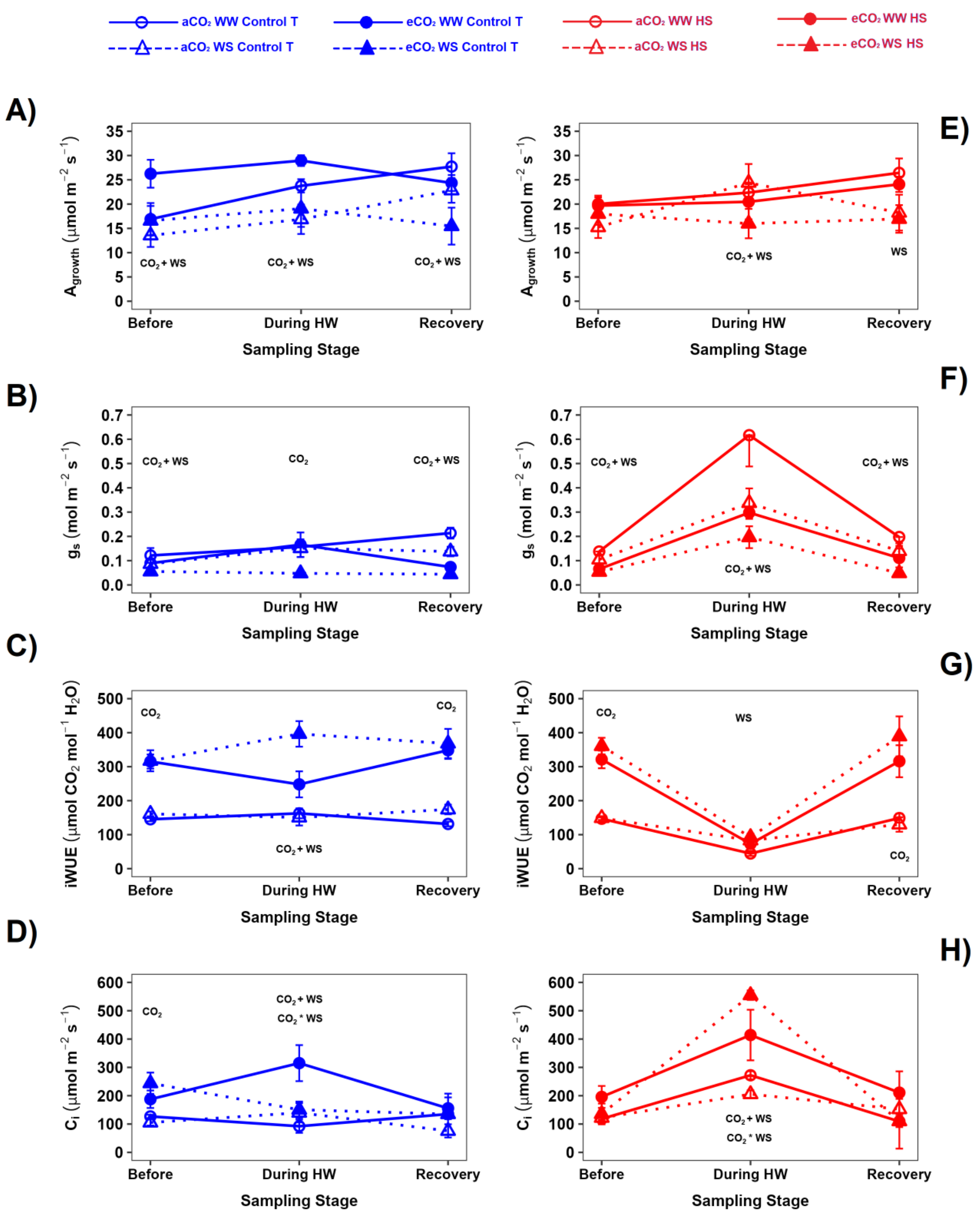

Fig.S2 Leaf gas exchange rates before, during and after the HS separated for the two sets of plants (one staying under control temperatures (blue) and the other experience a HS via a 6-day heat wave (red)). Open symbols= ambient [CO_2], Filled symbols= elevated [CO_2]; solid line=well-watered (WW), dashed line= water stressed (WS). Each point in the plot is the mean of both lines for that treatment combination (*n*=8; error bars=standard error). Statistical information in the insert are the result of a MANOVA (see Table S1). Treatments with "+" sign means both CO_2 and WS had an effect, while the "*" signals an interaction effect.

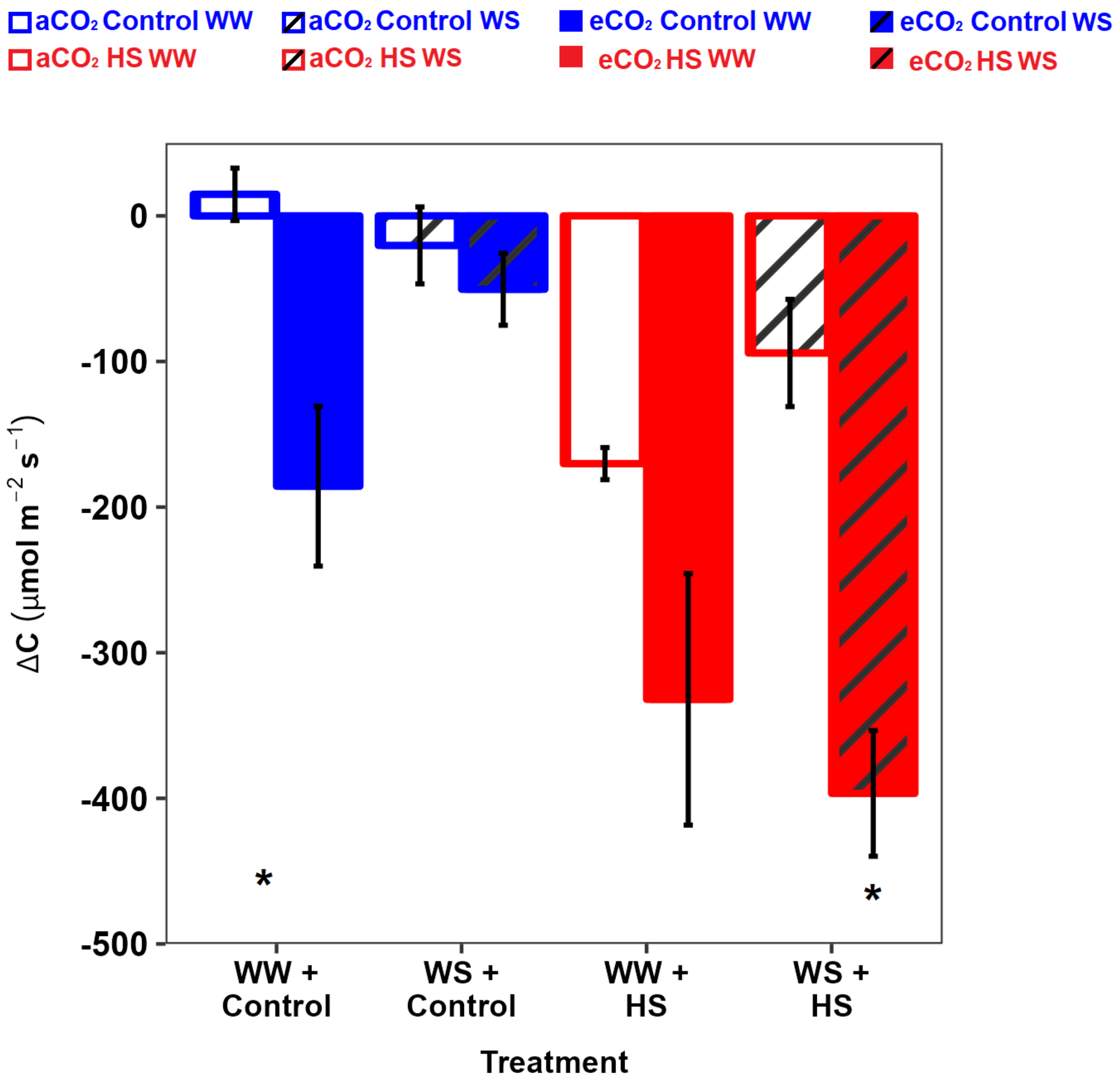

Fig.S3 Response of intercellular [CO_2] (*Ci*) differential (*ΔC*) (saturating C_i – operating C_i) to combined treatments of water (well-watered: WW; water-stress: WS), [CO_2] (400 ppm: aCO_2; 780 ppm: eCO_2) and extreme temperatures (31˚C: Control; 43˚C: heat-stress (HS)) in sorghum. See materials and methods for water stress, CO_2 and temperature treatment implementation, and for measurement conditions. Each bar chart represents the combined mean of the lines sampled at that treatment combination (*n*=8; error bars=standard error). Star symbol (*) represent statistically significant variation between the two [CO_2] treatments at $P<0.05$.

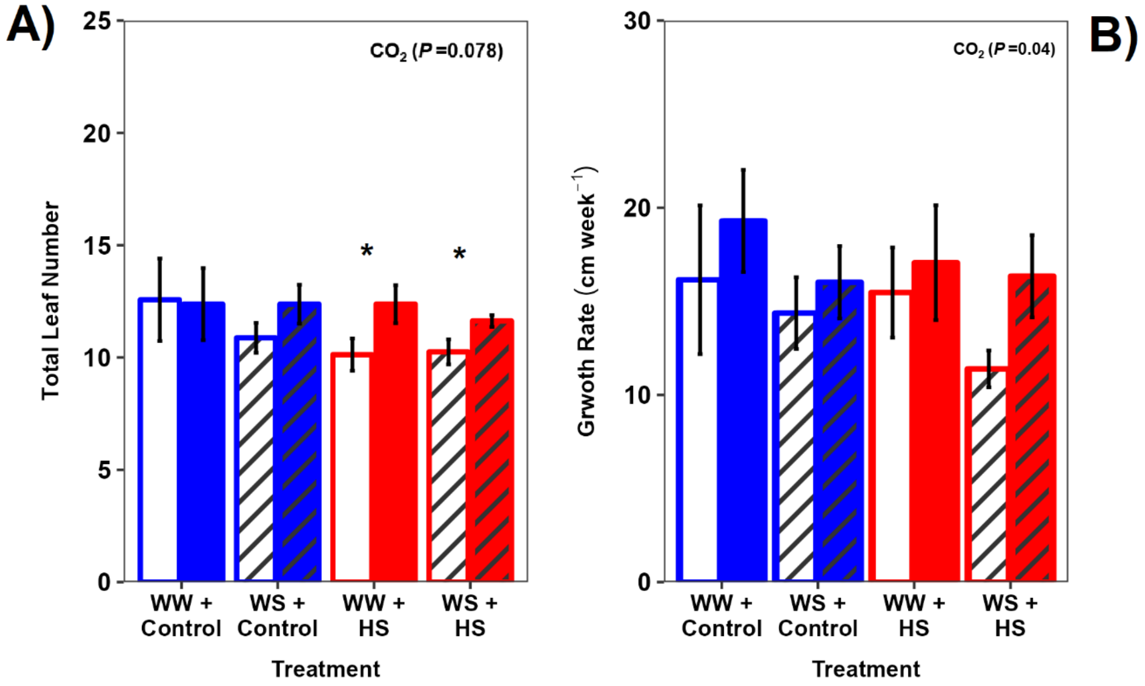

Fig.S4 Response of total leaf number at harvest and growth rate per week combined treatments of water (well-watered: WW; water-stress: WS), [CO_2] (400 ppm: aCO_2; 780 ppm: eCO_2) and extreme temperatures (31˚C: Control; 43˚C: heat-stress (HS)) in sorghum. See materials and methods for water stress, CO_2 and temperature treatment implementation, and for measurement conditions. Each bar chart represents the combined mean of the lines sampled at that treatment combination (n=8; error bars=standard error). Star symbol (*) represent statistically significant variation between the two [CO_2] treatments at P<0.05, while statistical information in the insert are the result of a MANOVA (see Table 1). For legend information, see Fig. S3.

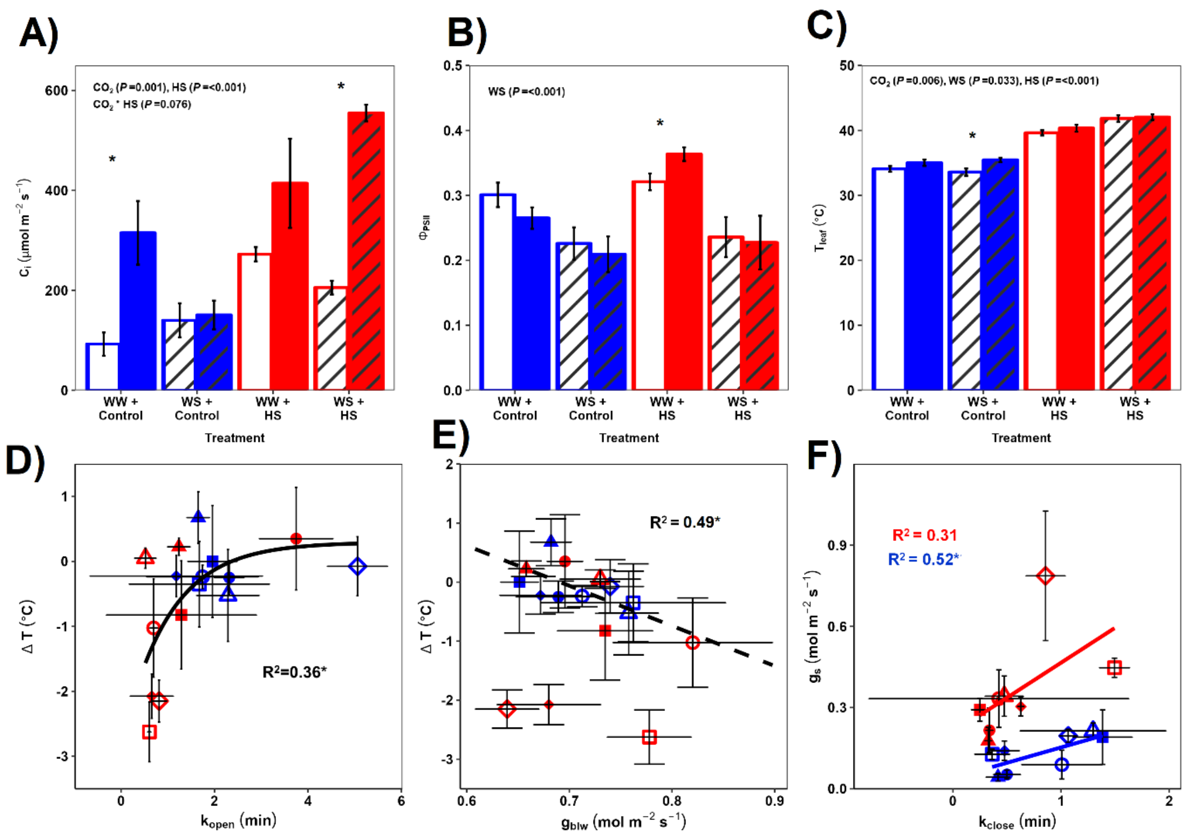

Fig.S5 (A-C) Response of leaf physiological factors combined treatments of water (well-watered: WW; water-stress: WS), [CO2] (400 ppm: aCO_2; 780 ppm: eCO_2) and extreme temperatures (31˚C: Control; 43˚C: heat-stress (HS)) in sorghum. See materials and methods for water stress, CO_2 and temperature treatment implementation, and for measurement conditions. (A-C) Each bar chart represents the combined mean of the lines sampled at that treatment combination (n=8; error bars=standard error). Star symbol (*) represent statistically significant variation between the two [CO_2] treatments at P<0.05, while statistical information in the insert are the result of a MANOVA (see Table 1). For legend information, see Fig. S3. (D-E) Lines represent the best fit through the data. In (D) R^2 is the adjusted R^2 from an exponential fit. in (E) the line is dashed because it represents the line of best fit after excluding ΔT =<-2). In (F) the red line indicates the fit through heat stress (HS) treatment and blue line through control). R^2 values are from a Pearson product-moment correlation analysis (n=4; error bars=standard error). (A) intercellular [CO_2] concentration (C_i); (B) quantum yield of photosystem II ($\Phi PSII$); (C) Leaf temperature (T_{leaf}); (D) Leaf-to-air temperature differential (ΔT) vs Kinetic rate constant of stomatal opening during transition from low-to-high light (k_{open}); (E) ΔT vs boundary layer conductance (g_{blw}) & (F) stomatal conductance (g_s) vs kinetic rate constant of stomatal closing during transition from high-to-low light (k_{close}). (For correlation plots: Control Temperatures=blue symbols; HS=red symbols; 400 ppm=open symbols, 780 ppm=filled symbols; well-watered + narrow leaf line= squares; well-watered + wide leaf line= rhombus; water-stress + narrow leaf line= circles; water-stress + wide leaf line= triangles. Dashed line represents the value at control conditions (400 ppm CO2, WW, control T).

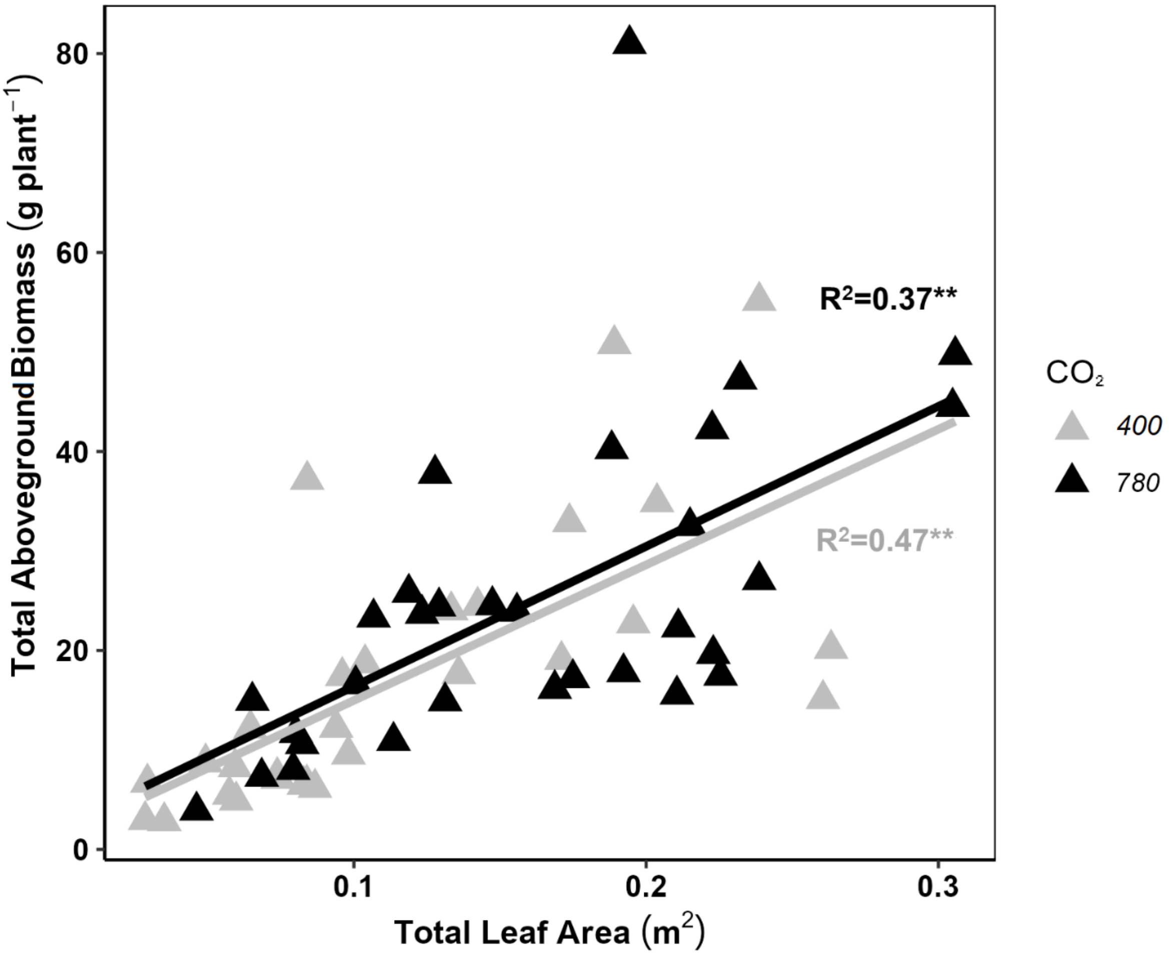

Fig.S6 Relationship between total leaf area (*TLA*) and total aboveground biomass (stem + leaf + panicle dry weight). *TLA* was calculated as leaf mass per area (*LMA*, g m^{-2}) divided by the dry mass of the harvested leaves (g). The plot shows the individual values for each replicate, with regressions separated by growth [CO_2].

REFERENCES

Buck AL. 1981. New equations for computing vapor pressure and enhancement factor. Journal of Applied Meteorology **20**, 1527–1532.

Marrero TR, Mason EA. 1972. Gaseous diffusion coefficients. Journal of Physical and Chemical Reference Data **1**, 3–118.

Nobel PS. 2009. *Physicochemical and Environmental Plant Physiology*. Burlington MA: Elsevier.

Robson TM, Sánchez-Gómez D, Cano FJ, Aranda I. 2012. Variation in functional leaf traits among beech provenances during a Spanish summer reflects the differences in their origin. Tree Genetics and Genomes **8**, 1111–1121.

Schlichting H, Gersten K. 2016. *Boundary Layer Theory*. Springer Berlin Heidelberg.

Sperry JS, Adler FR, Campbell GS, Comstock JP. 1998. Limitation of plant water use by rhizosphere and xylem conductance: results from a model. Plant, Cell and Environment **21**, 347–359.
